# Phosphate‐ and pH‐dependent self‐assembly of recombinant spider silk proteins

**DOI:** 10.1002/pro.70554

**Published:** 2026-04-06

**Authors:** Vanessa T. Trossmann, Veronika Hovanová, Tim Schiller, Martin Humenik, Erik Sedlák, Thomas R. Scheibel

**Affiliations:** ^1^ Department of Biomaterials, Faculty of Engineering Science University of Bayreuth Bayreuth Germany; ^2^ Laboratory of Functional Biointerfaces FZU – Institute of Physics of the Czech Academy of Sciences Prague Czech Republic; ^3^ Department of Biophysics, Faculty of Science P.J. Šafárik University Košice Slovakia; ^4^ Center for Interdisciplinary Biosciences, Technology and Innovation Park P.J. Šafárik University Košice Slovakia; ^5^ Department of Biochemistry, Faculty of Science P. J. Šafárik University Košice Slovakia; ^6^ Bayreuth Center for Colloids and Interfaces University of Bayreuth Bayreuth Germany; ^7^ Bayreuth Center for Molecular Biosciences University of Bayreuth Bayreuth Germany; ^8^ Bayreuth Center for Material Science University of Bayreuth Bayreuth Germany; ^9^ Bavarian Polymer Institute University of Bayreuth Bayreuth Germany

**Keywords:** kosmotropic ions, nanofibrils, particles, protein conformation, secondary structures

## Abstract

The process of molecular self‐assembly is an omnipresent mechanism in nature to generate a variety of efficient and functional hierarchical architectures, and inspires tailored material design and development. Thereby, self‐assembly is based on a controlled interplay and association of monomers into highly ordered structures triggered by different non‐covalent interactions. However, in the context of structural protein self‐assembly, this association process could be influenced by the underlying amino acid sequence as well as external triggers including pH value, protein concentration, or ionic composition. Thus, understanding their impact on protein conformation and assembly is indispensable for controlled protein processing and functional materials' engineering. Here, we analyzed the self‐assembly behavior of the intrinsically unstructured, recombinant spider silk proteins eADF4(Ω16) and eADF4(C16), which only differ in one amino acid residue in their repetitive module (glutamine and glutamic acid, respectively), depending on the concentration of kosmotropic potassium phosphate (KPi) and the pH value. The low protein charge in eADF4(Ω16) at neutral pH led to a compacted protein conformation and a significantly increased sensitivity to phosphate resulting in faster assembly kinetics of nanofibrils and precipitation of particles at lower KPi concentrations. In contrast, the presence of glutamic acid residues in eADF4(C16) enhanced the solubility and stability of protein monomers above physiological pH but led to an enhanced assembly/aggregation along with decreasing pH‐values. Interestingly, deprotonation of tyrosine residues at pH 10 introduced negative charges resulting in decreased hydrophobic interactions and thus decelerated restructuring and assembly of eADF4(Ω16). Our results enabled the identification of Pi‐ and pH‐dependent conformation and assembly models of eADF4‐based spider silk proteins allowing controllable processing into fibrils, particles, or hydrogels for specific applications.

## 1 INTRODUCTION

Protein self‐assembly is based on a controlled interaction between monomers into higher ordered structures allowing the efficient generation of hierarchical architectures in nature. The process of self‐assembly is triggered by different non‐covalent interactions including electrostatic, hydrophobic and van der Waals interactions as well as hydrogen bonding and aromatic stacking (Mendes et al., 2013; Philp & Stoddart, 1996; Roy & Pillai, 2023; Whitesides et al., 1991; Whitesides & Grzybowski, 2002). In this context, the fibril and fiber formation of structural proteins, such as collagen, elastin, keratin or silk, depends on repetitive amino acid (AA) sequences (peptide motifs) directing the non‐covalent interactions (Ferraro et al., 2016; Revell et al., 2021; Schiller & Scheibel, 2024; Solomonov et al., 2024). The respective protein sequence and/or structure requires specific stimuli, including pH, concentration, temperature and ionic conditions, for multiscale fibril formation (Hovanová, Hovan, Humenik, & Sedlák, 2023; Hovanová, Hovan, Žoldák, et al., 2023; Schiller & Scheibel, 2024; Zhu et al., 2018). For instance, collagen I contains the tri‐peptide repeats Gly‐X‐Y, where X and Y are mostly represented by proline and hydroxyproline residues, respectively. They are responsible for the formation of helical structures leading to triple‐helix assembly via hydrophobic interactions and further development of protofibrils, fibrils and fibers stabilized via intermolecular ionic and hydrogen bonding (Ferraro et al., 2016; Okuyama et al., 2012; Zhu et al., 2018).

In contrast, spider silk proteins comprise highly repetitive sequences with larger peptide modules (30–40 AA in case of major ampullate spidroins) that are intrinsically unstructured but fold into β‐sheets during the spidroins' assembly into solid fibers (Chan et al., 2020; Gosline et al., 1999; Gu et al., 2020; Humenik et al., 2011; Suzuki et al., 2022; Tokareva, Jacobsen, et al., 2014). Structural studies revealed that the AA composition and secondary structures of the spider silk proteins are mainly responsible for the mechanical fiber properties (Gosline et al., 1999; Hayashi et al., 1999; Holland et al., 2008; Parkhe et al., 1997; Simmons et al., 1994). Importantly, a pH drop from 8 to 5 (Andersson et al., 2014; Rising & Johansson, 2015; Yin et al., 2024), a simultaneous decrease of the concentration of chaotropic sodium chloride and the increase of kosmotropic potassium phosphate significantly influence β‐sheet formation of the spider silk proteins *in vivo* and *in vitro* (Hofer et al., 2012; Humenik et al., 2011; Jastrzebska et al., 2016; Knight & Vollrath, 2001; Lammel et al., 2008; Mohammadi et al., 2020; Oktaviani et al., 2019; Saric et al., 2021; Slotta et al., 2008; Yin et al., 2024). The molecular interactions within the fibers are based on hydrophobic stacking of the β‐sheets into nanocrystals, which are embedded in an H‐bonded amorphous matrix of unstructured regions containing β‐turns, β‐spirals and 3_1_‐helices (Glišović et al., 2008; Gosline et al., 1999; Humenik et al., 2011; Keten et al., 2010; Kiseleva et al., 2020; Lefèvre et al., 2007; Van Beek et al., 2002; Verma et al., 2021). On the molecular level, poly‐alanine‐rich stretches (Ala)_n_ in the repetitive core domains are self‐assembling into β‐sheets, while glycine‐ and proline‐rich protein stretches (e.g., GPGXY, GXX) represent an amorphous matrix (Hayashi et al., 1999; Humenik et al., 2011; Patil et al., 2022; Saric et al., 2021).

Besides structural proteins in which fibrillar structures are part of their biological function, fibril assembly is also crucial in terms of none‐structural proteins including amyloid proteins, for example, the amyloid precursor protein (APP) involved in various biological functions including neuronal development and signaling in its unassembled form, as well as Alzheimer's disease upon assembly (Viswanathan & Greenberg, 2011). During the non‐amyloidogenic pathway, APP is cleaved by α‐secretase, which could be enhanced by neuronal activity (Haass et al., 1995). In contrast, the amyloidogenic pathway involves sequential cleavage of APP by β‐secretase and γ‐secretase at the N‐ and C‐termini of the Aβ region, respectively (Joshi & Wang, 2015), resulting in the formation of the intrinsically disordered, peptide monomers Aβ40 (40 AAs) and Aβ42 (42 AAs), which can aggregate/assemble into soluble oligomers, protofibrils, and finally, insoluble amyloid fibrils, all displaying parallel or anti‐parallel β‐sheet structures (Agrawal & Skelton, 2019; Chen et al., 2017; Roychaudhuri et al., 2009; Yu et al., 2009). The Aβ fibril formation follows three‐step assembly kinetics containing primary nucleation, fibril elongation and secondary nucleation, catalyzed on the fibril surface similar to that identified for recombinant spider silk assembly (Dobson, 2003; Hovanová, Hovan, Humenik, & Sedlák, 2023; Humenik et al., 2014). Thereby, the primary nucleation and self‐association of Aβ monomers guided by molecular forces, for example, hydrophobic interactions and electrostatic effects, are pH‐dependent and accelerated at acidic conditions (Dobson, 2003; Tian & Viles, 2022). Moreover, seeding the reaction using pre‐formed fibril fragments bypasses the primary nucleation phase, making elongation and surface‐catalyzed secondary nucleation the dominant pathways (Hovanová, Hovan, Humenik, & Sedlák, 2023; Hovanová, Hovan, Žoldák, et al., 2023; Humenik et al., 2014; Humenik et al., 2015; Tian & Viles, 2022). Transmission electron microscopy (TEM) and atomic force microscopy (AFM) displayed the fibrillar morphology, while x‐ray diffraction, Fourier transform infrared spectroscopy (FTIR) as well as thioflavin‐T and Congo red binding revealed cross‐β‐sheet structures for Aβ fibrils (Nasica‐Labouze et al., 2015; Nilsson, 2004; Numata & Kaplan, 2011; Paravastu et al., 2006) as well as for recombinant spider silk‐based nanofibrils (Arndt et al., 2022; De Oliveira et al., 2023; Hovanová, Hovan, Humenik, & Sedlák, 2023; Hovanová, Hovan, Žoldák, et al., 2023; Humenik et al., 2014; Humenik et al., 2015; Numata & Kaplan, 2011; Oroudjev et al., 2002; Qi et al., 2023; Slotta et al., 2007), where β‐strands are stabilized by hydrogen bonds and oriented perpendicular to the fibril's long axis.

The recombinant spider silk protein eADF4(C16) (engineered *Araneus diadematus* fibroin 4) is inspired by the repetitive core domain of ADF4, one of at least two major ampullate spidroins of the European garden spider, and comprises 16 repeats of a consensus sequence (C‐module: GSSAAAAAAAASGPGGYGPENQGPSGPGGYGPGGP) (Huemmerich et al., 2004). Due to one glutamic acid residue in each repetitive unit, recombinant eADF4(C16) has a negative net charge at neutral pH (Huemmerich et al., 2004; Trossmann & Scheibel, 2023). In contrast to the natural spidroins, recombinant eADF4(C16) consists solely of a repetitive core domain, lacking the globular N‐ and C‐terminal domains (Saric et al., 2021; Trossmann & Scheibel, 2023). It is known that these terminal domains play a crucial role in natural spidroin self‐assembly and fiber spinning (Saric et al., 2021). There, the interaction of the terminal domains is necessary to form a spidroin network being a requirement for the final fiber assembly (Schiller & Scheibel, 2024). Including the terminal domains in recombinant variants is beneficial for increasing the protein's solubility and for mimicking liquid–liquid phase separation as found in the natural fiber spinning process (Saric et al., 2021; Schiller & Scheibel, 2024). However, for the use of non‐native particles and nanofibrils in material science applications, only the recombinant spider silk proteins resembling the repetitive core domain are required, whereas the terminal domains are in this case rather detrimental. In this context, like other recombinant spider silk proteins and peptides (Numata & Kaplan, 2011; Oroudjev et al., 2002; Qi et al., 2023), recombinant eADF4‐variants are able to self‐assemble into stable nanofibrils via hydrophobic interactions without the presence of the terminal domains. (Hovanová, Hovan, Humenik, & Sedlák, 2023; Hovanová, Hovan, Žoldák, et al., 2023; Humenik et al., 2014; Humenik et al., 2015; Kumari et al., 2020; Lechner et al., 2022; Neubauer et al., 2021; Trossmann et al., 2022).

**FIGURE 1:**
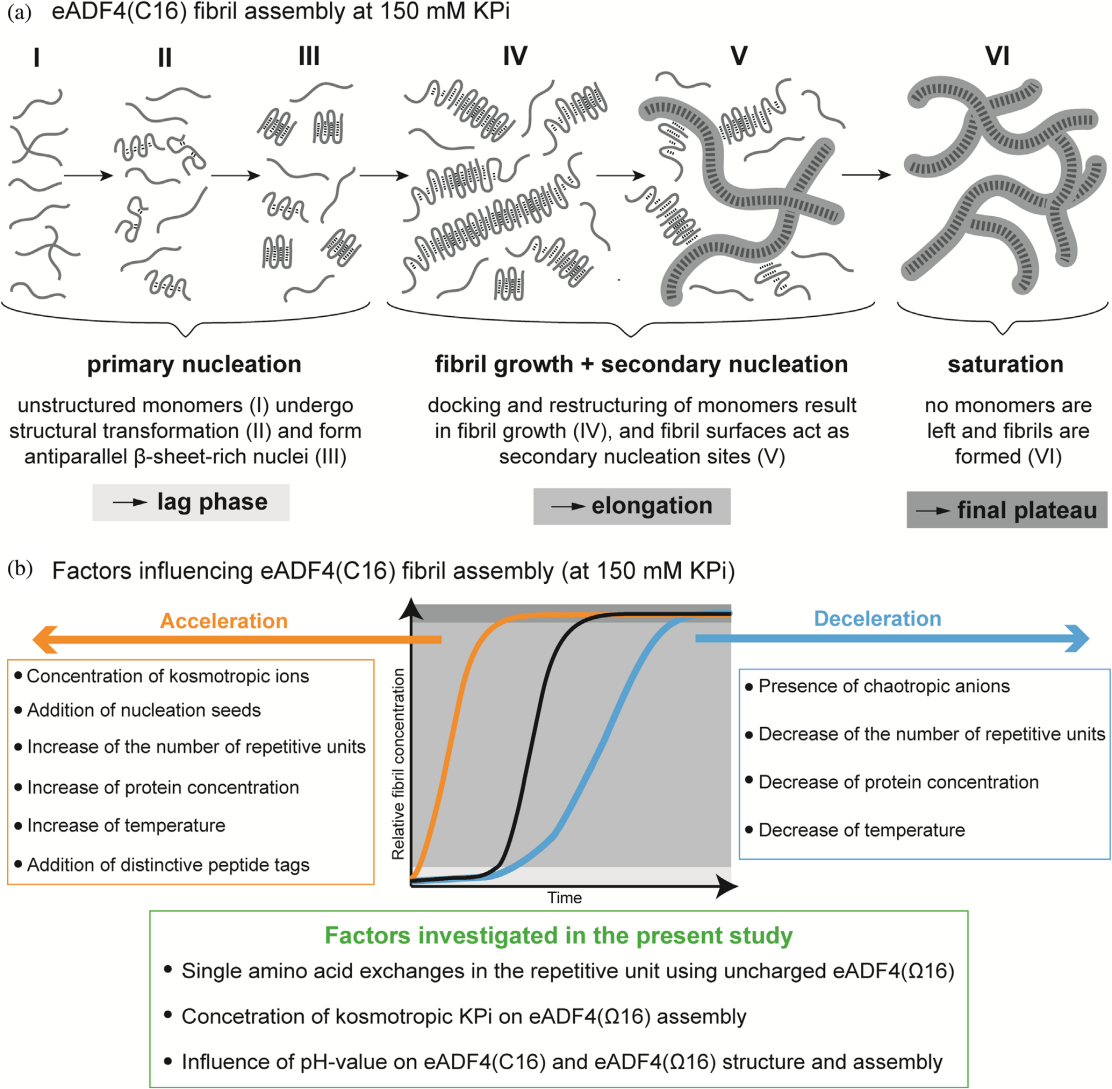
Factors influencing eADF4(C16) spider silk fibril self‐assembly. (a) eADF4(C16) follows a nucleation‐dependent self‐assembly process for fibril formation in presence of kosmotropic anions (e.g., Pi). In the primary nucleation phase (lag phase), intrinsically unstructured monomers (I) undergo structural transformation (anti‐parallel β‐sheets) (II) forming β‐sheet‐rich nuclei (seeds) via hydrophobic interactions (III). During the fibril elongation phase, additional monomers attach to these nuclei (seeds) and undergo restructuring, leading to fibril growth and extension (IV). Moreover, secondary nucleation occurs, as monomers interact with the surface of existing fibrils, accelerating fibril formation and promoting secondary nucleation (V). In the saturation phase (final plateau), fibril formation is completed (VI) (Hovanová, Hovan, Žoldák, et al., 2023; Humenik et al., 2014). (b) Summary of factors that accelerate (orange) or decelerate (blue) eADF4(C16) fibril formation (Hovanová, Hovan, Humenik, & Sedlák, 2023, Hovanová, Hovan, Žoldák, et al., 2023, Humenik et al., 2014, Humenik et al., 2015, Lechner et al., 2022, Slotta et al., 2008) including factors that are investigated in this study (green). The black curve illustrates a typical eADF4(C16) assembly curve at RT and a protein concentration of 1 mg/mL in presence of 150 mM KPi.

Furthermore, previous studies indicated that kosmotropic phosphate ions (Pi) at concentrations below 300 mM trigger self‐assembly of eADF4(C16) into nanofibrils at physiological pH, while Pi concentrations above 400 mM induce particle formation (Hovanová, Hovan, Humenik, & Sedlák, 2023; Hovanová, Hovan, Žoldák, et al., 2023; Humenik et al., 2014; Humenik et al., 2015; Lammel et al., 2008; Slotta et al., 2007; Slotta et al., 2008; Wehr et al., 2026). It was shown that eADF4(C16) nanofibril formation follows a primary nucleation‐dependent assembly mechanism (Humenik et al., 2014; Humenik et al., 2015; Humenik & Scheibel, 2014) and comprises an additional secondary nucleation pathway (Hovanová, Hovan, Žoldák, et al., 2023) (Figure 1a). Importantly, the assembly kinetics of the engineered variants are influenced by the number of repetitive units, protein concentration, presence of nucleation seeds, nature and concentration of kosmotropic ions, temperature, and presence of additional, distinctive peptide tags at the protein's termini (Figure 1b) (Hovanová, Hovan, Humenik, & Sedlák, 2023; Hovanová, Hovan, Žoldák, et al., 2023; Humenik et al., 2014; Humenik et al., 2015; Lechner et al., 2022; Slotta et al., 2008). Changes in environmental conditions could be used to affect the characteristics of the fibrils (Hovanová, Hovan, Humenik, & Sedlák, 2023; Hovanová, Hovan, Žoldák, et al., 2023) as well as to translate the self‐assembly of the proteins into diverse applications on surfaces (Heinritz et al., 2022; Molina et al., 2019) or in bulk, yielding hydrogels (Desimone et al., 2017; Lechner et al., 2022; Neubauer et al., 2021; Schacht & Scheibel, 2011; Trossmann et al., 2022).

A point mutation in the repetitive module allowed an exchange of glutamic acid to glutamine residues yielding the variant eADF4(Ω16) with zero net charge at neutral conditions (Kumari et al., 2020; Trossmann & Scheibel, 2023). Correspondingly, when lysine residues are introduced, the positively charged counterpart eADF4(κ16) is achieved (Trossmann & Scheibel, 2023). Interestingly, studies on hydrogel formation based on experiences with fibril self‐assembly in the case of eADF4(C16) (Schacht & Scheibel, 2011) showed significant changes in gelation kinetics and gel stability in the case of eADF4(Ω16) and eADF4(κ16) (Neubauer et al., 2021). For instance, positively charged eADF4(κ16) showed a stronger tendency to aggregation compared to eADF4(C16) and eADF4(Ω16) under similar aqueous conditions, and required specific conditions to induce protein assembly and fibril formation (Neubauer et al., 2021). Therefore, we only compared herein eADF4(C16) with eADF4(Ω16) to elucidate the influence of kosmotropic phosphate ions and pH under aqueous conditions on the conformational changes and self‐assembly behavior of the recombinant spider silk proteins (Figure 1b).

## 2 RESULTS AND DISCUSSION

### 2.1 Influence of kosmotropic potassium phosphate on eADF4(Ω16) at neutral pH

**FIGURE 2:**
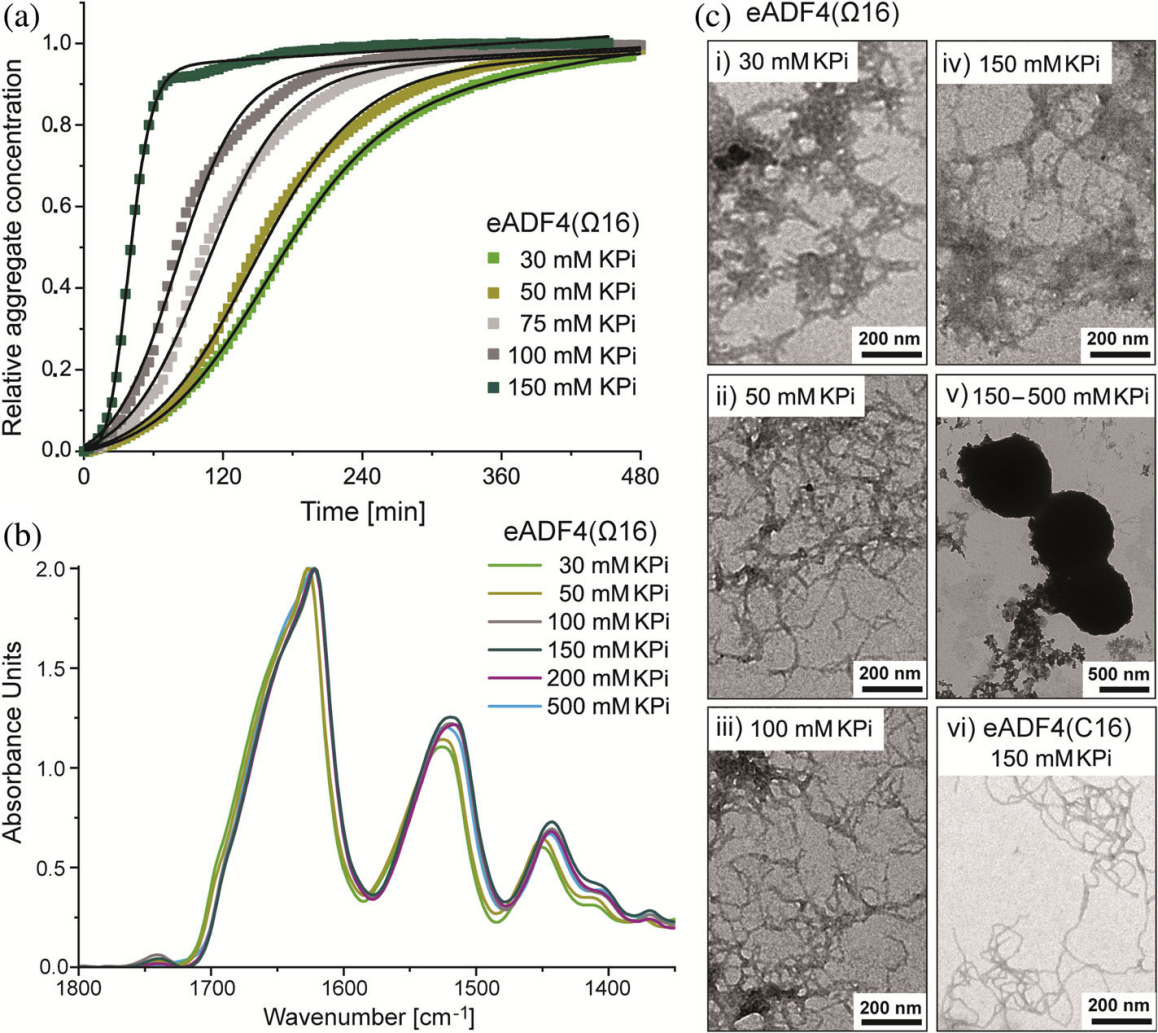
Influence of potassium phosphate (KPi) on self‐assembly of eADF4(Ω16) without charged amino acid residues. (a) Normalized turbidity evolution of eADF4(Ω16) at 340 nm indicating increasing self‐assembly rates with increasing KPi concentrations. Data were normalized, averaged over three replicates at each condition (*n* = 3) and fitted to sigmoidal curves (Equation 1). (b) FTIR‐spectra of the amide I, II and III regions indicate β‐sheet‐rich secondary structure content of the assembled eADF4(Ω16) structures verified by FSD analysis (Table S2). (c) TEM showing formation of eADF4(Ω16) nanofibrils between 30 mM (i) and 150 mM KPi (iv) and particle formation beginning at 150 mM KPi (v). For comparison, TEM images of eADF4(C16) nanofibrils obtained at 150 mM KPi (vi) are shown.

The influence of different KPi concentrations (up to 500 mM KPi, pH 8) on eADF4(Ω16) assembly has been investigated (Figure 2) in comparison to that on the well‐established eADF4(C16) at 150 mM (Figure S1). Turbidity measurements at 340 nm revealed a higher sensitivity of eADF4(Ω16) toward kosmotropic Pi than of eADF4(C16) (Figure 2a vs. Figure S1), administered in shorter lag times and higher apparent rate constants (Table S1A vs. C).

As shown by the kinetic data (Table S1A), an acceleration of eADF4(Ω16) assembly was observed with increasing KPi concentrations (from 30 mM to 150 mM). Hence, at 150 mM KPi, self‐assembly of eADF4(Ω16) was already finished within 2 h, while for eADF4(C16), the final plateau, indicating consumption of the monomers, was reached only after 33 h (Figure S1, pH 8) at the same protein concentration (Hovanová, Hovan, Humenik, & Sedlák, 2023; Hovanová, Hovan, Žoldák, et al., 2023; Humenik et al., 2014; Humenik et al., 2015). UV–Vis spectroscopy at 280 nm confirmed complete depletion of the spider silk monomers in the supernatant after self‐assembly as shown previously (data not shown) (Humenik et al., 2015; Slotta et al., 2008). Thus, the shorter lag phases of eADF4(Ω16) indicated a faster nucleation compared to eADF4(C16) (see also Figure 1a).

After reaching the final plateau, the eADF4(Ω16) protein assemblies were investigated using FTIR‐spectroscopy and Fourier‐Self‐Deconvolution (FSD) of the amide I band (Hu et al., 2006) to determine secondary structure content (Figure 2b, Table S2) and TEM to analyze morphology (Figure 2c). FTIR‐spectra indicated β‐sheet‐rich secondary structure for all eADF4(Ω16) protein assemblies independent of the KPi concentration, since the amide I bands showed maxima at wavenumbers at around 1630 cm^−1^ (Figure 2b). This observation was verified by FSD analysis of the amid I bands indicating a β‐sheet content higher than 40% (Table S2).

TEM analysis allowed to distinguish between aggregates, particles and nanofibrils (Figure 2c, Figure S2), revealing that eADF4(Ω16) self‐assembled into fibrils between 30 and 150 mM KPi (Figure 2c, i‐iv, Figure S2A–D, i) corresponding with the kinetic data and the sigmoidal turbidity increase (Figure 2a). However, the fibril morphology varied significantly dependent on the KPi concentration. At 30 mM KPi, only short, “molten” fibrils were visible, which clustered into bigger aggregates (Figure 2c,i, Figure S2A). Probably both pathways, the KPi‐induced nucleation and self‐assembly of eADF4(Ω16) and the protein aggregation occurring commonly without KPi, were present in that case. However, 50 and 100 mM KPi supported the self‐assembly pathway resulting in long fibrils with typical entanglements (Figure 2c, ii‐iii, Figure S2B,C). Hence, this KPi concentration range seemed to support the formation of seeds, ordered interaction of soluble eADF4(Ω16) thereon and fibril elongation. Interestingly, at 150 mM KPi, only short, molten and clustered fibrils were visible, resembling those at 30 mM KPi (Figure 2c, iv, Figure S2D, i). Furthermore, TEM images revealed an alternative pathway leading to formation of particles, which became dominating above 150 mM KPi (Figure 2c,v, Figure S2D, i–H). Figure S2 shows characterization of the spherical eADF4(Ω16) particles at KPi‐concentrations between 150 mM and 500 mM using TEM. In addition, TEM and SEM images showing spherical eADF4(Ω16) nano‐particles precipitated at 1 M KPi are displayed in Figure S3. To sum up, eADF4(Ω16) is able to form long, entangled, branched fibrils in the concentration regime of 50–100 mM KPi showing faster assembly kinetics than eADF4(C16). The fibrils are comparable to that of eADF4(C16) (exemplarily shown for 150 mM KPi in Figure 2c, vi), although the KPi concentration range for nucleation and fibril formation was significantly broader (10–300 mM) (Hovanová, Hovan, Humenik, & Sedlák, 2023; Hovanová, Hovan, Žoldák, et al., 2023; Humenik et al., 2014; Humenik et al., 2015; Slotta et al., 2008). The transition zone, where both, fibrils and particles, were observed, was at 400 mM KPi in case of eADF4(C16), and particles could only be precipitated above 500 mM (Lammel et al., 2008; Slotta et al., 2008). These results are consistent with a recent study by Wehr et al., who showed that eADF4(C16) nanofibrils and particles reveal high structural and dynamic similarities at the atomic level using solid‐state NMR analyses, although both morphologies differed substantially in their macroscopic appearance (Wehr et al., 2026).

**FIGURE 3:**
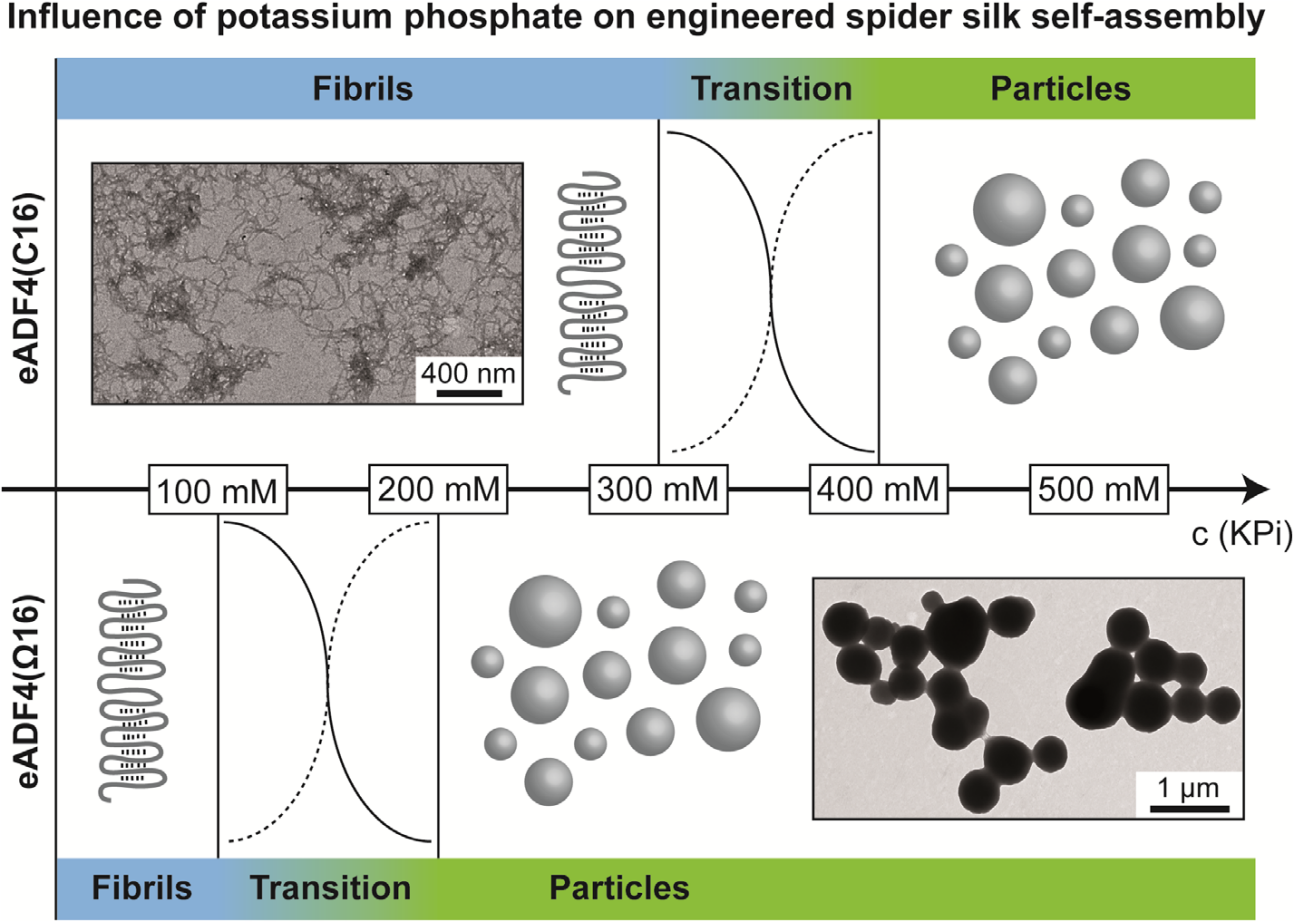
Impact of KPi concentration on self‐assembly of two different recombinant eADF4 variants into nanofibrils and particles. Since eADF4(Ω16) is more sensitive toward KPi compared to eADF4(C16) (Slotta et al., 2008), precipitation into particles can already be detected at a KPi concentration of 150 mM. Thus, the transition zone, where nanofibrils and particles are formed and coexist, is at lower KPi concentrations in case of eADF4(Ω16) (100–200 mM).

Considering these findings, a sequence‐dependent assembly model of engineered eADF4‐based proteins was created (Figure 3). Negatively charged eADF4(C16) shows high hydration and stability in aqueous buffers after dialysis when staring with chaotropic solvents at RT due to electrostatic repulsion, since the theoretical isoelectric point (IEP) is at 3.5. Zeta potential measurements in KCl (pH 2.5–10.0) and isoelectric focusing revealed experimental IEPs of around 3.9 and 4.5, respectively (Lentz et al., 2022). Since eADF4(C16) contains glutamic acid residues, it is able to form non‐covalent interactions with the solvent and buffer components via its carboxyl groups and stays as an unstructured monomer in solution at RT. Thus, eADF4(C16) shows a low aggregation and slow assembly behavior in the absence of any trigger (e.g., addition of KPi or shear forces), administered by its solution stability over several weeks. eADF4(Ω16) with its glutamine residues (i.e., pH‐independent uncharged residue) showed time‐, temperature‐, and concentration‐dependent protein aggregation in buffers at pH values between 7 and 8 (Kumari et al., 2020; Neubauer et al., 2021). At RT, eADF4(Ω16) precipitation was significantly accelerated, indicated by an increase in turbidity and occurrence of opaque assemblies/aggregates after several hours, even without an external trigger. Moreover, eADF4(Ω16) showed lower critical solution temperature (LCST) behavior, being stabilized in solution at 4°C.

A possible explanation for the pronounced aggregation of the protein without typical triggers, such as kosmotropic ions or shear forces, could be that the IEP of eADF4(Ω16) with 7.7 (Lentz et al., 2022) is inside the selected pH range of the used buffer systems. Isoelectric focusing of eADF4(Ω16) revealed an IEP between 7.8 and 8.3, and zeta potential measurements in KCl (pH 2.5–10.0) showed that the zeta potential was close to zero between pH 6.0 and 8.5 (Lentz et al., 2022). Since eADF4(Ω16) is uncharged, it shows reduced non‐covalent interactions with ions in aqueous buffers leading to an increase of hydrophobic protein–protein interactions and faster protein aggregation. It was shown previously that glutamic acid, aspartic acid and serine residues increase the solubility of proteins (e.g., RNAse) significantly, while hydrophilic AAs including glutamine, threonine and asparagine residues contribute unfavorably to protein solubility (Trevino et al., 2007).

At self‐assembly conditions (pH 7–8), the addition of kosmotropic phosphate to eADF4(C16) solutions decreases the hydration of the protein and allows a restructuring of the flexible protein backbone leading to increased intramolecular interactions (Hovanová, Hovan, Humenik, & Sedlák, 2023; Humenik et al., 2014). Hence, the poly‐alanine‐stretches form stable β‐sheets leading to meta‐stable seeds, which interact with further soluble protein monomers, finally directing fibril elongation (Hovanová, Hovan, Žoldák, et al., 2023; Humenik et al., 2014; Wehr et al., 2026). With increasing KPi concentrations, the hydration decreases further, leading to enhanced intramolecular protein–protein interactions and salting‐out effects resulting in spider silk particle precipitation (Humenik et al., 2015; Jastrzebska et al., 2016; Krishnaji et al., 2014; Kucharczyk et al., 2018; Lammel et al., 2008; Peng & Wen, 2025; Qi et al., 2023; Slotta et al., 2008; Tokareva, Lin, et al., 2014; Yu et al., 2024). Therefore, below 300 mM KPi eADF4(C16) spider silk monomers self‐assemble into nanofibrils, while particles precipitate at salt concentrations above 400 mM KPi. Interestingly, in the transition zone between 300 and 400 mM KPi, both species were formed and coexist (Lammel et al., 2008, Slotta et al., 2008).

In contrast, since eADF4(Ω16) is uncharged at neutral pH, low to no electrostatic repulsion occurs. Therefore, less amounts of KPi are necessary to support protein restructuring and formation of hydrophobic protein interactions, leading to faster structure formation of eADF4(Ω16), nucleation and self‐assembly (Figure 2a). In addition, the lack of intermolecular protein repulsion made it more “salt‐sensitive,” shifting the transition midpoints, where nanofibrils and particles coexist, from 350 mM in case of eADF4(C16) to 150 mM KPi in case of eADF4(Ω16) (Figure 3). Furthermore, aggregation appeared at 30 and 150 mM KPi for eADF4(Ω16), which has not been detected in case of KPi‐triggered eADF4(C16) assembly. Hence, we presumed different conformations at the beginning of assembly of both proteins, due to significant charge differences (see below).

### 2.2 Hydrodynamic radii and conformation of spider silk proteins at neutral pH

**FIGURE 4:**
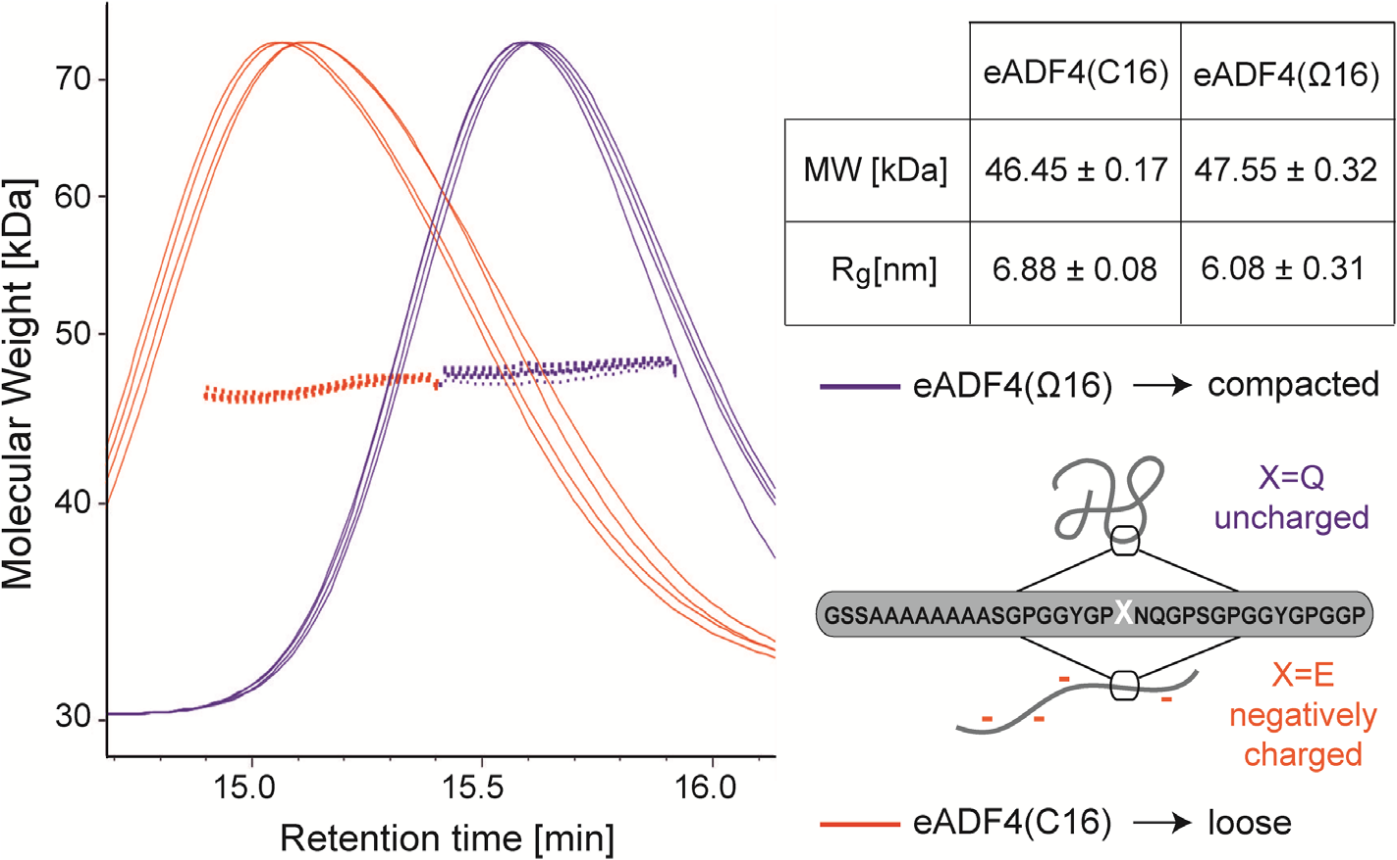
SEC‐MALS analyses revealed different hydrodynamic radii of eADF4(C16) and eADF4(Ω16). Negatively charged eADF4(C16) showed lower retention times indicating larger hydrodynamic radii (*R*_*H*_) as well as larger calculated *R*_*g*_ compared to the later retention times (smaller *R*_*H*_) and smaller calculated *R*_*g*_ of the uncharged eADF4(Ω16). Data were obtained from four independent measurements for each protein (*n* = 4). Differences in the calculated MWs from the SEC‐MALS data were caused by the uncertainty of the MALS calculations resulting from the difference in applied protein concentrations (3 and 1 mg/mL for eADF4(C16) and eADF4(Ω16), respectively). MALDI‐TOF confirmed similar MWs (see Figure S3).

We further analyzed the soluble spider silk variants in low ionic strength buffer to gain more insights in the starting protein conformation at neutral pH. Far‐UV CD‐spectra confirmed that monomers of both proteins were intrinsically unstructured concerning their secondary structure in this state (after dialysis and ultracentrifugation), as observed previously (Huemmerich et al., 2004; Humenik et al., 2014; Trossmann & Scheibel, 2023) (Figure S4). To confirm the hypothesis that the presence of charged AAs influences the hydrodynamic radius of a protein and, hence, inter‐ and intramolecular interactions during assembly events, SEC‐MALS was conducted (Figure 4).

Since both proteins have the same number of AAs (*n* = 576) and similar molecular weights (MW = 47.7 kDa) confirmed by MALDI‐TOF analysis (Figure S5), an apparent difference in retention time observed for eADF4(C16) (~15 min, Figure 4, orange curve) and eADF4(Ω16) (~15.7 min, Figure 4, purple curve) at the same elution conditions strongly indicated different conformational packing of the proteins. This was supported by the calculated geometric radii (*R*_*g*_ = 6.88 nm for eADF4(C16) and *R*_*g*_ = 6.08 nm for eADF4(Ω16)). The larger geometric radius of negatively charged eADF4(C16) resulted most probably from its intramolecular repulsion, that is, loosely packed, unstructured conformation. In contrast, the smaller geometric radius of eADF4(Ω16) points toward a more compact protein structure (Figure 4). Upon addition of KPi (Humenik et al., 2014; Humenik et al., 2015) or increasing protein concentrations, eADF4(C16) formed fibrils below 10 mg/mL and physically crosslinked fibrillar networks, that is, hydrogels above 10 mg/mL (Desimone et al., 2017; Kumari et al., 2020; Lechner et al., 2022; Neubauer et al., 2021; Schacht & Scheibel, 2011; Trossmann et al., 2022). In case of eADF4(Ω16), uncharged glutamine residues allow compaction of the proteins supporting intramolecular hydrophobic interaction of poly‐alanine motives. This enhances their transition into beta‐sheets and, consequently, formation of nuclei toward aggregation and assembly, depending on the presence of KPi. These results are in line with a previous study showing that eADF4(Ω16) is stabilized upon addition of amphiphilic dimethylsulfoxid (DMSO) as co‐solvent, most probably shielding the hydrophobic polyalanines and increasing the interaction with the surrounding solvent by formation of hydrogen bonds (Neubauer et al., 2021). Furthermore, solid‐state NMR analyses of eADF4(C16) nanofibrils prepared at pH 8 showed that all glutamic acid residues were deprotonated implying their localization on the outside of the fibril, where the negative charge can be compensated (Wehr et al., 2026).

### 2.3 Influence of pH on eADF4 assembly and aggregation

**FIGURE 5:**
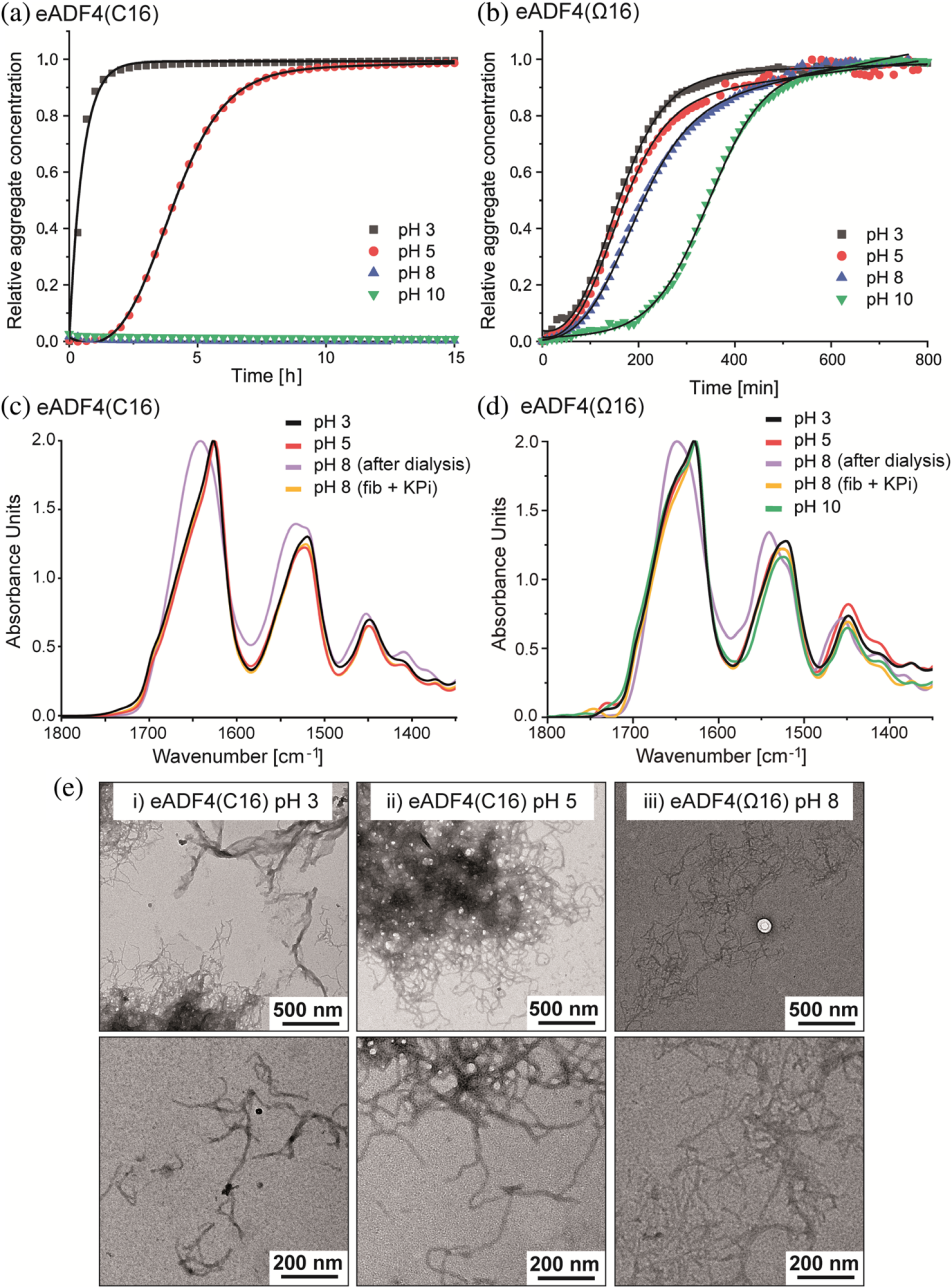
Analysis of the pH‐dependent assembly/aggregation behavior of eADF4 variants. Turbidity measurements at 340 nm indicated pH‐dependent assembly kinetics of eADF4(C16) (a) and pH‐independent kinetics of eADF4(Ω16) (b). FTIR spectra of assembled protein species showed β‐sheet‐rich assemblies in the case of eADF4(C16) (c) as well as in the case of eADF4(Ω16) (d). For comparison, FTIR spectra of protein fibrils were included, formed in the presence of KPi at pH 8 (150 mM for eADF4(C16) and 100 mM for eADF4(Ω16)). (e) TEM images revealed fibril assembly of eADF4(C16) at pH 3 and pH 5 as well as of eADF4(Ω16) at pH 8.

Since charge and polarity of AAs of the protein backbone showed impact on the hydration status, colloidal stability, conformation and self‐assembly of eADF4, we further analyzed assembly/aggregation behavior of eADF4(C16) and eADF4(Ω16) at different pH values (pH 3–10) to gain detailed insights in (de‐)protonation effects. Consecutive protonation of glutamic acid residues in eADF4(C16), that is, its charge neutralization, transferred the behavior of this protein to an eADF4(Ω16)‐like one, as confirmed by turbidity kinetics at 340 nm at different pH values (Figure 5). The dropping pH affected solubility and self‐assembly/aggregation of eADF4(C16) much stronger (Figure 5a) than of eADF4(Ω16) (Figure 5b). eADF4(C16) remained soluble at a pH ≥6 but displayed highly accelerated assembly/aggregation at pH ≤5, showing similar kinetic parameters as eADF4(Ω16) (Table S1B) most probably due to its low pI. Extensive protonation of the glutamic acid residues led to a charge distribution resembling uncharged eADF4(Ω16) with its glutamine residues. The importance of charges is stressed in the behavior of eADF4(Ω16), being substantially less sensitive to pH changes in comparison to eADF4(C16) and showing similar assembly/aggregation kinetics between pH 3 and 9 (Figure 5b and Table S1B).

To confirm the role of protein charge as well as of the presence of soluble protein (after dialysis), we induced additional charges on tyrosine residues by increasing the pH above a value of 8. For eADF4(Ω16), the assembly/aggregation kinetics of eADF4(Ω16) were slowed down at pH 10 (Figure 5b). Since eADF4(C16) was stable in solutions at pH ≥6 (Figure 5a), we analyzed the self‐assembly kinetics at higher pH values (pH 8–10) in presence of 150 mM KPi to induce fibril formation (Figure S1). In this context, differences in assembly kinetics are solely influenced by the changed pH value, as phosphate is mainly 2‐fold negatively charged between pH 7 and 12 (Han, 2020). At pH 10, the kinetics showed a significant extension of the lag time (5‐fold), that is, slower formation of oligomers, whereas fibril growth kinetics (elongation phase) were more similar to those at pH 8 and 9 (2‐fold increase) (Figure S1 and Table S1C).

Studies based on molecular modeling, NMR‐spectroscopy and photometric titrations demonstrated that tyrosine residues and their specific location within repetitive modules (including motifs like GPGGY found in eADF‐variants) are indicators of a pre‐structured state in solution (Stengel et al., 2023). It is speculated that pre‐structuring, driven by π–π stacking and CH/π interactions, promotes the formation of beta‐sheet structures during assembly (Chalek et al., 2024; Chen et al., 2024; Dicko et al., 2004). A recent solid‐state NMR study indicated that the tyrosine residues in eADF4(C16) assemblies are rigidified suggesting their engagement in π–π stacking interactions (Wehr et al., 2026). However, in solutions with a pH of 10, spider silk proteins based on ADF4 sequences contained solvent‐exposed tyrosine residues (pKa ~10) due to partial negative charges (Dicko et al., 2004; Oktaviani et al., 2018; Oktaviani Nur et al., 2012). Hence, deprotonation diminishes tyrosine‐based intramolecular interactions of the eADF4‐variants at pH 10. Therefore, the structure is somewhat loosening, which is reducing the probability of hydrophobic intermolecular interactions and β‐sheet formation based thereon, that is, it inhibits to some extent nucleus formation. A similar sensitivity toward acidification can be seen in the self‐assembly of amyloid fibril‐forming histidine‐rich Aβ monomers showing an isoelectric point pI of 5.3. Above the pI, deprotonated, negatively charged histidine residues increase the peptides solubility. However, with decreasing pH, the histidine residues become protonated leading to a simultaneous decrease of electrostatic repulsion between the AA chains and increase of intramolecular, hydrophobic interactions (Tian & Viles, 2022).

For both eADF4‐based variants, the protein assemblies at different pH values were analyzed using FTIR spectroscopy and Fourier‐Self‐Deconvolution (FSD) of the amide I band (Hu et al., 2006) to determine secondary structure content (Figure 5 C‐D, Table S3, Figures S6 and S7). The symmetrical peak of eADF4(C16) at 1650 cm^−1^ indicated mainly random coil secondary structure at pH 8 (Figure 5c, lilac curve), as verified by FSD (Table S3, Figure S6). In contrast, the amide I bands of assemblies formed at pH 3 and pH 5 showed maxima at 1630 cm^−1^ and appeared overlapping with the spectrum of nanofibrils classically formed in the presence of 150 mM KPi at pH 8 (Figure 5c). FSD‐analysis also confirmed an increased β‐sheet content (>40%) of eADF4(C16) assemblies at pH 3, pH 5 and in presence of 150 mM KPi, while random coil and α‐helices were decreased compared to soluble eADF4(C16) at pH 8 (Table S3, Figure S6).

Similar results were observed for eADF4(Ω16) (Figure 5d, Table S3, Figure S7). At pH 8 (Figure 5d, purple curve), eADF4(Ω16) also exhibited mainly random coil secondary structure after ultracentrifugation, as indicated by the symmetrical peak at 1650 cm^−1^ and confirmed by FSD analysis (Table S3, Figure S7). In addition, asymmetrical amide I bands of assemblies formed at pH 3, pH 5, and pH 10 exhibited their maxima at around 1630 cm^−1^ and appeared overlapping with the spectrum of nanofibrils formed in the presence of 100 mM KPi at pH 8 (Figure 5d). FSD‐analysis verified an increased β‐sheet content (>39%) of eADF4(Ω16) assemblies at pH 3, pH 5, pH 10 and in the presence of 100 mM KPi, with the maximum β‐sheet value for fibrils formed at 100 mM KPi, while random coil structures and α‐helices were decreased compared to soluble eADF4(Ω16) at pH 8 (Table S3, Figure S7). However, the more pronounced shoulders around 1650 cm^−1^, in comparison to eADF4(C16) in Figure 5c, also refer to higher random coil and α‐helical content, as confirmed by FSD (Table S3, Figure S7). Interestingly, at pH 8, soluble eADF4(C16) displayed a significantly lower β‐sheet content (14%) than eADF4(Ω16) (23%, Table S3). Hence, the simultaneous increase in β‐sheet content and decrease in random coil structure confirmed the enhanced pre‐structuring and compaction of eADF4(Ω16) at pH 8 in comparison to the more loosely packed and unstructured eADF4(C16) already seen in SEC‐MALS (Figure 4), which is responsible for accelerated fibril formation and aggregation of eADF4(Ω16).

The morphology of eADF4‐based assemblies formed at different pH‐values was analyzed using TEM enabling the distinction between guided assembly into nanofibrils and uncontrolled protein aggregation. TEM images revealed that the β‐sheet‐rich eADF4(C16)‐assemblies at lower pH‐values (pH 3 and 5) were mainly ordered fibrils and their conglomerates, and only few, bulky aggregates (Figure 5e,i‐ii). Therefore, no additional kosmotropic phosphate ions are necessary to induce fibril formation below pH 5. However, eADF4(C16) fibril length decreases with dropping pH. The reason could be the occurrence of accompanying aggregation depleting the monomers' availability for fibril growth as well as the increase of nucleation events, that is, more docking sites for eADF4(C16) monomers. TEM images also verified that eADF4(Ω16) assembles into ordered fibrils, as exemplarily shown for samples harvested at pH 8 without addition of KPi (Figure 5e, iii), whereas eADF4(C16) stayed soluble under these conditions. In this context, the morphology and length of eADF4(C16) fibrils at pH 3 (Figure 5e,i) likely resemble those of eADF4(Ω16) fibrils at pH 8 (Figure 5e, iii), since both proteins showed identical net charge and accelerated nucleation and assembly behavior under these conditions.

Taken together, the pH‐dependent aggregation analysis of eADF4(C16) and eADF4(Ω16) confirmed the conclusions drawn by analyzing the SEC‐MALS data (Figure 4) and displayed that eADF4(Ω16) is already pre‐structured, shows a similar charge distribution, and high level of restructuring at all investigated pH‐values due to intra‐ and intermolecular interactions. In contrast, eADF4(C16) is deprotonated, soluble, and elongated above neutral pH and needs external triggers, such as pH‐drop or kosmotropic phosphate, to initiate conformational changes.

**FIGURE 6:**
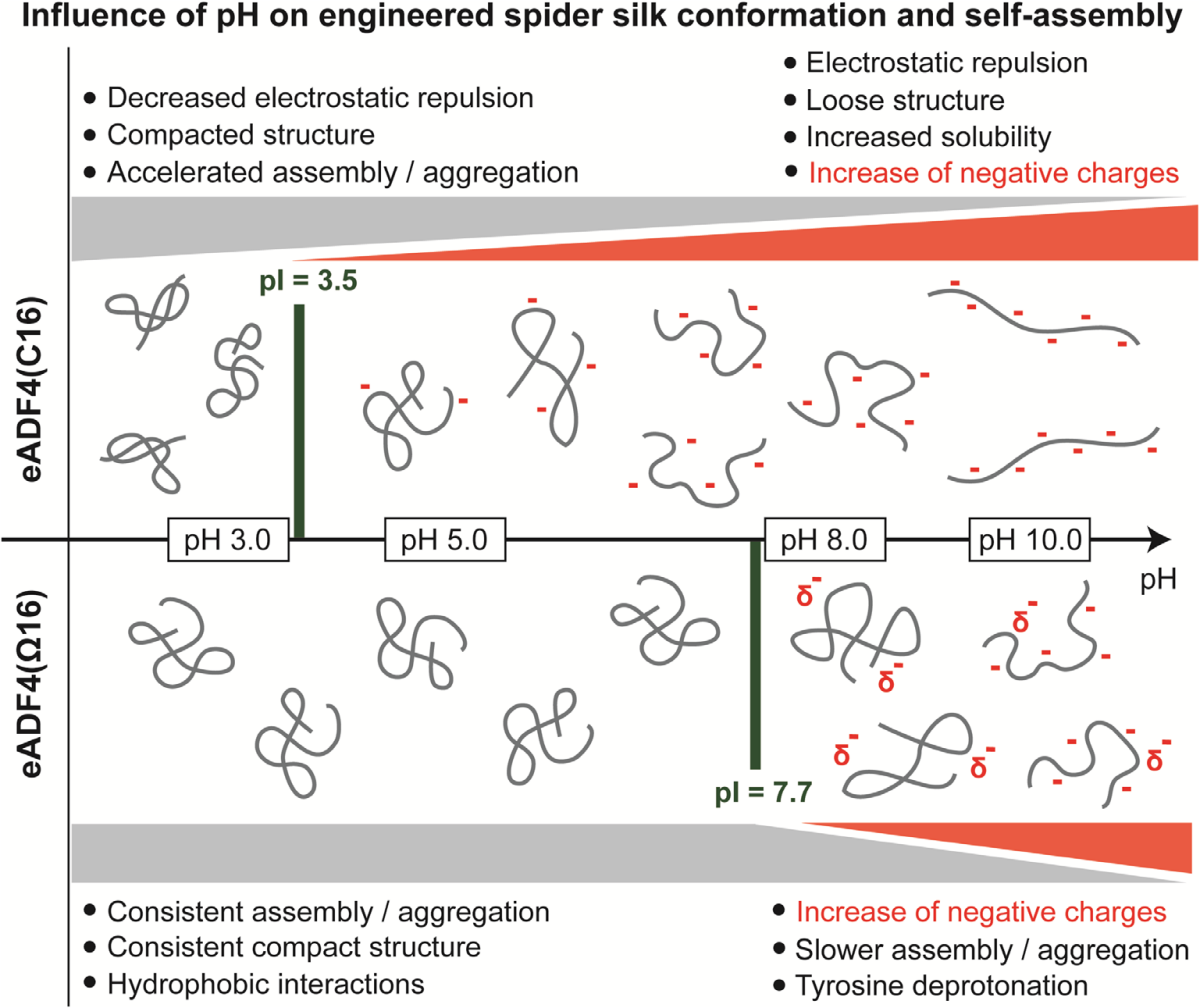
Schematic model of the protein conformation of eADF4(C16) and eADF4(Ω16) at various pH values explaining their different assembly behavior due to deprotonation of tyrosine residues resulting in an increase of negative charges, as well as protonation of glutamic acid residues in eADF4(C16).

Based on these results and in addition to the previous sequence‐dependent model, a pH‐dependent structure and self‐assembly model for eADF4(C16) and eADF4(Ω16) has been developed (Figure 6) highlighting the correlation of pH, charge, and protein conformation. In general, a pH‐drop and the associated protonation and change in charge influence the conformation of eADF4(C16) much stronger than that of the uncharged eADF4(Ω16).

Above physiological pH, eADF4(C16) shows high solubility and an expanded, less compact structure due to electrostatic repulsion of the glutamic acid residues in the repetitive modules. With decreasing pH, an increased restructuring and compaction of eADF4(C16) occurred due to increased protonation and decreased electrostatic repulsion. Below the IEP of eADF4(C16) (IEP = 3.5), the complete loss of electrostatic repulsion led to maximum compaction of monomeric eADF4(C16), resulting in accelerated assembly/aggregation. Due to the complete protonation of the carboxyl groups at pH 3 and the accompanying loss of charges, the strong hydration and solvent interaction of eADF4(C16) is suddenly diminished. Combined with the removal of electrostatic repulsion, the protein backbone collapses and restructures quickly, leading to enhanced intramolecular protein–protein interactions accelerating self‐assembly and aggregation. In contrast, uncharged eADF4(Ω16) has time to get accustomed to a polar solvent during dialysis. Thus, pH changes don't affect hydration and conformational state. Therefore, conformation and assembly/aggregation behavior of eADF4(Ω16) are scarcely influenced by pH shifts, as there are no significant changes in (de‐)protonation or protein charge. This uncharged protein consistently shows a compacted protein structure causing continuous assembly/aggregation based on hydrophobic interactions over a wide pH range. However, the deprotonation of tyrosine residues (two per repetitive C‐/Ω‐module) at pH 10 introduces local negative charges, which increase the hydration status, the interaction with the solvent, and the electrostatic repulsion of the protein backbone. Thus, the structure of eADF4(Ω16) is not strictly compacted anymore, resulting in decelerated restructuring and less intramolecular protein–protein interactions leading to fewer nucleation events as well as decreased assembly rates and fibril growth.

## 3 CONCLUSION AND OUTLOOK

**FIGURE 7:**
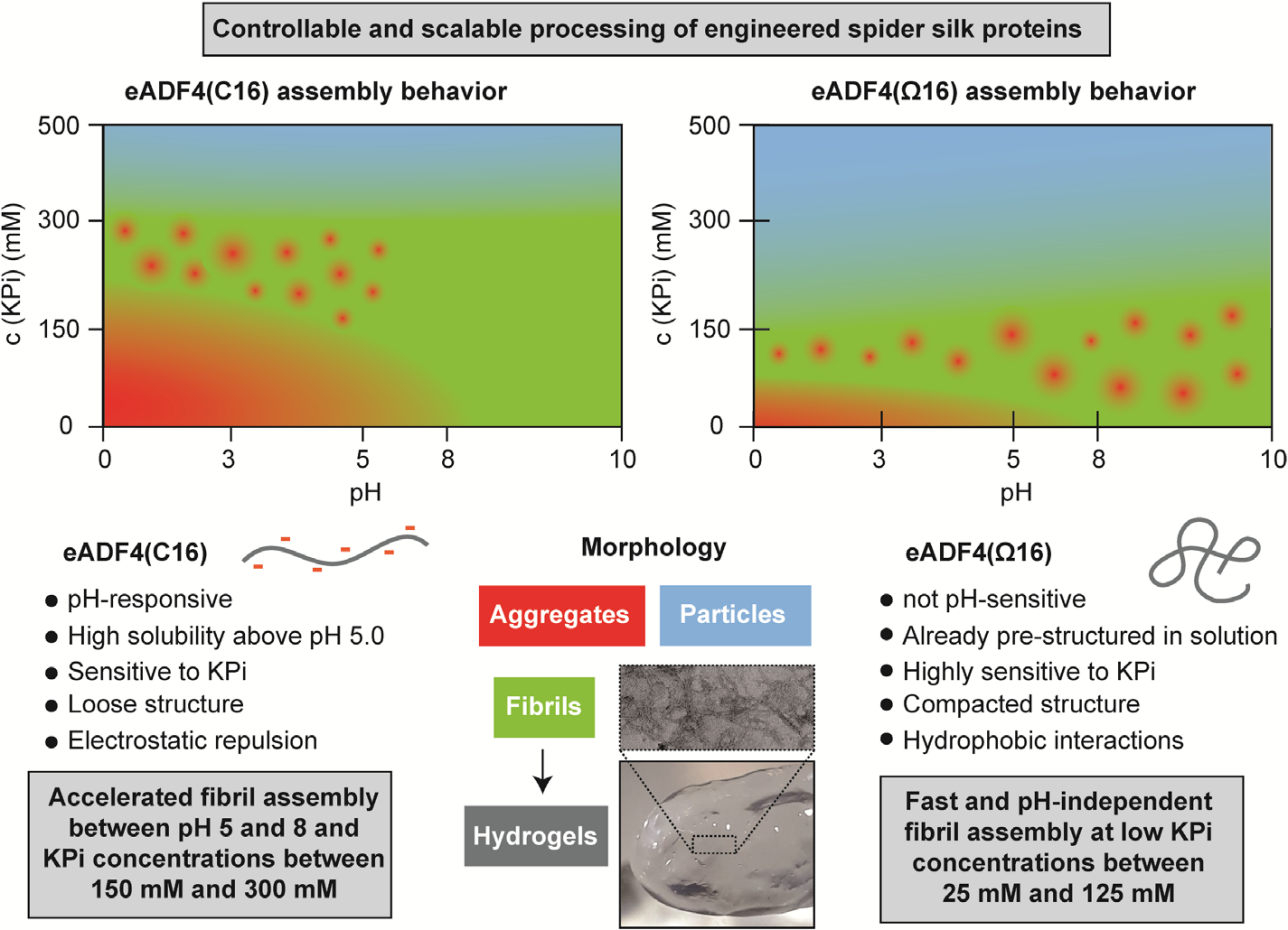
Identification of optimal conditions for recombinant spider silk processing. By unraveling critical stimuli influencing recombinant spider silk protein assembly, processing conditions for spider silk materials could be predicted, optimized, and adjusted.

We could show that the exchange of negatively charged AAs (glutamic acid residues) to uncharged equivalents (glutamine ones) altered solubility and conformation at physiological pH from a completely soluble, loosely packed, elongated state (eADF4(C16)), to a pre‐structured, compacted state (eADF4(Ω16)), respectively, as well as the sensitivity toward kosmotropic phosphate and pH‐shifts (Figure 7). We identified that protein restructuring and guided fibril formation could be controlled and scaled by adopting external conditions including the concentration of kosmotropic phosphate or the pH‐value, but their influence differs significantly between both proteins. Based on our findings, we propose a model how pH‐value and phosphate concentration could be potentially combined to find optimal processing conditions of engineered spider silk proteins (Figure 7).

Our results indicated that the pre‐structuring of eADF4(Ω16) monomers led to a shortened lag phase of fibril assembly indicating faster primary nucleation and a higher number of β‐sheet‐rich seeds. However, deprotonation of tyrosine residues at pH 10, and, thus, introduction of partial negative charges decreases hydrophobic interactions and restructuring. Therefore, our results expand the possibilities of processing artificial proteins in a targeted manner for specific purposes and applications (Figure 7). The processing conditions have to be protein‐specifically adjusted, since the processing parameters could significantly differ after exchanging even as little as just one AA in the repetitive module. Furthermore, since nanofibrils are also a route toward (hydro‐)gel networks, an in‐depth understanding of processing conditions and self‐assembly kinetics could increase the control over nucleation, assembly, and thus, gelation speed. The optimal gel formation is highly important for the generation of cell‐laden gels and bioinks. A fast gelation process ensures optimal cell distribution inside the silk scaffolds as well as high survival of encapsulated cells (Arndt et al., 2023; Desimone et al., 2017; Lechner et al., 2022; Trossmann et al., 2022). In conclusion, this study clearly demonstrated, how protein design and engineering could influence spider silk protein properties and self‐assembly behavior, allowing for the optimized, controllable and scalable processing of silk materials for technical or biomedical applications, tissue engineering, and biofabrication.

## 4 MATERIALS AND METHODS

### 4.1 Reagents and solutions

Experiments at different pH values were performed in the pH range from 3 to 10 with the following buffers at a concentration of 50 mM: pH 3 (Glycine‐HCl), pH 4 and 5 (sodium acetate‐NaOH), pH 6 (PIPES‐NaOH), pH 7–9 (TRIS‐HCl), and pH 10 (Glycine‐NaOH). Ultrapure water (Milli‐Q‐system) was used. If not otherwise stated, all chemicals were purchased from Carl Roth (Karlsruhe, Germany).

### 4.2 Protein solubilization and characterization

The engineered spider silk protein eADF4(C16) was purchased from AMSilk GmbH (Germany) as lyophilized powder. The uncharged eADF4(Ω16) variant was produced, purified, and analyzed as previously described (Kumari et al., 2020; Trossmann & Scheibel, 2023). The lyophilized proteins were dissolved in 6 M guanidinium thiocyanate and dialyzed overnight using dialysis membranes with a molecular weight (MW) cutoff of 6–8 kDa (Spectra/Por, Thermo Fisher Scientific GmbH, Germany). While eADF4(C16) was dialyzed against 5–10 mM Tris/HCl, pH 8 at room temperature, dialysis of eADF4(Ω16) was performed using 5–10 mM Tris/HCl, pH 8 at 4°C to avoid protein aggregation (Trossmann & Scheibel, 2023). Precipitates formed during dialysis were removed by ultracentrifugation (Optima MAX‐XP, Beckman‐Coulter, USA) at 185,000*g* at 4°C for 50 min. Protein concentrations were determined using an UV–VIS spectrophotometer (NanoDrop 1000, Thermo Fisher, USA). In the following, the protein solutions were characterized at different states to determine solubility and assembly/aggregation behavior: (i) immediately after ultracentrifugation at pH 8, (ii) after ultracentrifugation and addition of different potassium phosphate concentrations (30–500 mM KPi), (iii) after ultracentrifugation and addition of buffers with different pH values (pH 3–10), (iv) after incubation in buffers with various phosphate concentrations or at different pH values.

### 4.3 Far‐UV circular dichroism (CD) spectroscopy

Far‐UV CD spectroscopy was conducted between 200 and 250 nm to determine the secondary structure content of soluble, ultracentrifuged (1 h, 4°C, 55,000 rpm) spider silk proteins as reported previously (Hovanová, Hovan, Humenik, & Sedlák, 2023; Hovanová, Hovan, Žoldák, et al., 2023; Humenik et al., 2014; Trossmann & Scheibel, 2023). CD spectra were recorded on a J‐815 CD spectrometer (Jasco, Japan) using concentrations of 0.2 mg/mL, cuvettes with a path length of 0.1 cm, an integration time of 2 s, and a scanning speed of 100 nm min^−1^. Five spectra were recorded and averaged.

### 4.4 Matrix assisted laser desorption ionization with time‐of‐flight (MALDI‐TOF) mass spectroscopy

MALDI‐TOF molecular weight analysis was performed as previously described (Trossmann & Scheibel, 2023). The protein solutions were treated with ZipTip C4 pipette tips (Millipore, Germany) to desalt and concentrate the proteins. Sinapinic acid‐matrix (20 mg/mL in 60% acetonitrile and 0.1% trifluoroacetic acid) was used for elution and spotting on a ground steel target plate. Spectra were recorded using a Bruker AutoFlex Max mass spectrometer equipped with a Smartbeam II laser (Bruker, Germany) in linear mode and an acceleration voltage of 20 kV.

### 4.5 Size exclusion chromatography coupled to multiangle light scattering (SEC‐MALS)

SEC‐MALS was conducted using a Superdex 200 Increase 10/300 GL‐column (Cytiva, Germany) connected to an Agilent 1100 Series HPLC system with a MALS detector DAWN EOS (Wyatt, Germany) as reported previously (Humenik & Scheibel, 2014) to determine the molecular weights and protein sizes. Ultracentrifuged eADF4(C16) and eADF4(Ω16) protein solutions at concentrations of 1–3 mg/mL were applied via a 200 μL loop at a flow rate of 0.8 mL/min. The eluted proteins were analyzed using a UV‐detector at 280 nm, a refractive index detector Shodex RI‐71 and a static light scattering detector DOWN EOS equipped with a K5 flow cell. Molecular masses and sizes were calculated from light scattering signals using the ASTRA 6 software (Wyatt, Germany). Data were obtained from four independent measurements for each protein.

### 4.6 Measurements of self‐assembly/aggregation kinetics

Ultracentrifuged protein solutions (10 μM) were monitored every 10 min at 27°C using turbidity measurements at 340 nm in a plate reader (SpectraMax iD5, Molecular Devices, Germany) or a UV‐spectrometer (Cary 50 UV, Varian, Germany), following the addition of potassium phosphate at various concentrations (30–500 mM KPi, pH 8) or buffers with different pH values (pH 3 (Glycine‐HCl), pH 4 and pH 5 (sodium acetate‐NaOH), pH 6 (PIPES‐NaOH), pH 7–9 (TRIS‐HCl) and pH 10 (Glycine‐NaOH)) to determine assembly/aggregation kinetics. For analyzing eADF4(C16) fibril formation between pH 8, 9, and 10 under similar conditions, 150 mM KPi (2‐fold negatively charged phosphate) were added to induce self‐assembly. To analyze the amount of remaining non‐assembled spider silk monomers in the supernatant after ultracentrifugation, UV–Vis spectroscopy at 280 nm was conducted after running self‐assembly/aggregation kinetics as shown previously (Humenik et al., 2015; Slotta et al., 2008).

### 4.7 Fitting the kinetic data using a sigmoidal curve

Turbidity measurements were plotted as a function of time and fitted by a sigmoidal curve using Equation 1 and Origin (OriginLab Corporation) (Equation 1) (Nielsen et al., 2001) as described previously (Humenik et al., 2014).

(1) $$y=y_{i}-m_{i}x+\frac{y_{f}-m_{f}x}{1+e^{\frac{-x-x_{0}}{\tau }}}$$

There, $y_{i}-m_{i}x$ represents the initial slope during the lag phase, $y_{f}-\mathrm{xt}$ is the slope after the growth phase, and $x_{0}$ represents the time at 50% conversion. The apparent rate constant, *k*_app_, for the growth of fibrils is given by 1/τ, and the lag time is given by *x*_*o*_ *− 2τ* (Table S1). This method enables comparison of lag times in different fibril assembly kinetics (Nielsen et al., 2001).

### 4.8 Fourier transform infrared (FT IR) spectroscopy

FTIR spectra were recorded on a Bruker Tensor 27 (Ettlingen, Germany) using an attenuated total reflection (ATR) sampling technique on a germanium crystal. Spider silk proteins and their assemblies were characterized in a solid state after lyophilization. Spectra were recorded in absorbance mode at RT, with a 4 cm^−1^ resolution in the range of 900–4000 cm^−1^. OPUS software (version 6.5, Bruker Optik, GmbH) was used to process the data (corrected for background and atmosphere). Only Amide I‐III range (1300–1800 cm^−1^) spectra with information on protein secondary structure are shown. Fourier self‐deconvolution (FSD) of the Amide I band was carried out as reported previously (Hu et al., 2006). Data were averaged from three FSD evaluations for each protein.

### 4.9 Transmission electron microscopy (TEM)

TEM was conducted as described previously (Hovanová, Hovan, Humenik, & Sedlák, 2023; Hovanová, Hovan, Žoldák, et al., 2023; Humenik et al., 2014; Neubauer et al., 2021; Trossmann et al., 2022). In brief, spider silk protein suspensions and assemblies were deposited on Pioloform‐carbon‐coated 200‐mesh copper grids (Plano GmBH, Germany) and incubated for 60–120 s at room temperature. Afterwards, the solution was blotted using a fiber free paper (Kimtech Kimwipes, Kimberly‐Clark), and the adsorbed samples were washed with water. After negative staining using 2% uranyl acetate for 60 s, the samples were washed again with water. The samples were dried for at least 24 h and stored in darkness until further use. Images were recorded using a Zeiss LEO EM922 Omega microscope (Zeiss Microscopy, Jena, Germany). The microscope was operated at 80 kV accelerating voltage. Images were recorded by a bottom mounted CCD camera system (Ultrascan 1000, Gatan, München, Germany) and processed with a digital imaging processing system (Digital Micrograph GMS 1.9, Gatan, München, Germany).

### 4.10 Scanning electron microscopy (SEM)

Washed spider silk particle solutions (0.4 mg/mL) were pipetted on silica wafers and dried at ambient conditions to allow solvent evaporation. Afterwards, the wafers were fixed on SEM stubs (Plano gmbH, Germany), surrounded by aluminum tape and sputter‐coated with platinum yielding a 2 nm layer using a Leica EM ACE 600 sputter coater (Leica, Germany). SEM analysis was conducted on a Thermo Scientific (FEI) Apreo VS device equipped with a field emission gun and a SE2‐detector operating at 2 kV (Thermo Fisher Scientific, Germany) as described previously (Trossmann et al., 2022).

## AUTHOR CONTRIBUTIONS

**Vanessa T. Trossmann**: Conceptualization; investigation; formal analysis; visualization; writing—original draft. **Veronika Hovanová**: Conceptualization; investigation; formal analysis; writing, reviewing and editing the draft. **Tim Schiller**: Investigation; formal analysis; reviewing and editing the draft. **Martin Humenik**: Conceptualization; formal analysis; funding acquisition; reviewing and editing the draft; supervision. **Erik Sedlák**: Funding acquisition; reviewing and editing the draft, supervision. **Thomas R. Scheibel**: Conceptualization; funding acquisition; reviewing and editing the draft; supervision.

## CONFLICT OF INTEREST STATEMENT

Thomas R. Scheibel is co‐founder and shareholder of AMSilk GmbH. The authors declare that they have no known competing financial interests or personal relationships that could have appeared to influence the work reported in this paper.

## Supporting information


**Table S1.** Corresponding apparent rate constants (k_app_) and lag times of kinetic measurements. (A) concerning the data shown in Figure 2a for 10 μM eADF4(Ω16) spider silk proteins in presence of different KPi concentrations; (B) concerning the data shown in Figure 5a for 10 μM eADF4(C16) and eADF4(Ω16) spider silk proteins in buffers with different pH values; (C) concerning the data shown in Figure S1 for 10 μM eADF4(C16) in KPi at indicated pH.
**Table S2.** Secondary structure content of recombinant eADF4(Ω16) assemblies at different KPi concentrations determined using FSD of FTIR‐spectra (Figure 1b) according to Hu et al. (2006). Data were averaged from three FSD evaluations for each sample (*n* = 3).
**Table S3.** Secondary structure content of recombinant spider silk assemblies at different pH conditions or soluble monomers directly after ultracentrifugation (bold) determined using FSD of FTIR‐spectra (Figures S5 and S6) according to Hu et al. (2006). For comparison, secondary structure contents of eADF4(C16) fibrils formed at pH 8 in presence of 150 mM KPi were included. Data were averaged from three FSD evaluations for each protein (*n* = 3).
**Figure S1.** Influence of pH on self‐assembly kinetics of eADF4(C16) in presence of 150 mM KPi adjusted to the indicated pH values (2‐fold negatively charged phosphate). Normalized turbidity evolution at 340 nm indicated decreasing self‐assembly rates and increasing lag times with increasing pH values. Data were averaged over three replicates at each condition (n = 3). Obtained dependencies were fitted using sigmoidal curves (Equation 1).
**Figure S2.** Influence of potassium phosphate (KPi) on self‐assembly of eADF4(Ω16) analyzed using TEM. The investigated KPi‐concentrations were (A) 30 mM, (B) 50 mM, (C) 100 mM, (D i + ii) 150 mM, (E) 200 mM, (F) 300 mM, (G) 400 mM and (H) 500 mM. TEM showed formation of eADF4(Ω16) nanofibrils between 30 mM and 150 mM KPi and particle formation beginning at 150 mM KPi. The orange box highlights the presence of both species, nanofibrils and particles, at a KPi‐concentration of 150 mM.
**Figure S3.** TEM (A) and SEM (B) of air‐dried, spherical eADF4(Ω16) particles precipitated at 1 M KPi using a salting‐out procedure.
**Figure S4.** Far‐UV circular dichroism spectra indicate a random coil secondary structure of soluble eADF4(C16) and eADF4(Ω16) after ultracentrifugation. Data were averaged over five replicates at each condition (*n* = 5).
**Figure S5.** Maldi‐ToF spectra confirm the theoretical MW of recombinant eADF4(C16) (theoretical MW: 47,698 Da) and eADF4(Ω16) (theoretical MW: 47,683 Da).
**Figure S6.** Exemplary FSD‐analysis of the Amide I band of eADF4(C16)‐based assemblies formed at pH 3 (A) and 5 (B), soluble protein at pH 8 (C) and fibrils formed in presence of 150 mM KPi (D) to determine secondary structure content (*n* = 3).
**Figure S7.** Exemplary FSD‐analysis of the Amide I band of eADF4(Ω16)‐based assemblies formed at pH 3 (A), 5 (B) and 10 (E), soluble protein at pH 8 (C) and fibrils formed in presence of 100 mM KPi (D) to determine secondary structure content (n = 3).

## Data Availability

The data that support the findings of this study are available from the corresponding author upon reasonable request.
