# Supplementary material for: Phosphate‐ and pH‐dependent self‐assembly of recombinant spider silk proteins
Source: Protein Sci. 2026 Apr 6;35(5):e70554. doi: 10.1002/pro.70554 (PMC13051846; doi:10.1002/pro.70554)
Supplement: Supplementary file 1 — Table S1. Corresponding apparent rate constants (kapp) and lag times of kinetic measurements. (A) concerning the data shown in Figure 2a for 10 μM eADF4(Ω16) spider silk proteins in presence of different KPi concentrations; (B) concerning the data shown in Figure 5a for 10 μM eADF4(C16) and eADF4(Ω16) spider silk proteins in buffers with different pH values; (C) concerning the data shown in Figure S1 for 10 μM eADF4(C16) in KPi at indicated pH. Table S2. Secondary structure content of recombinant eADF4(Ω16) assemblies at different KPi concentrations determined using FSD of FTIR‐spectra (Figure 1b) according to Hu et al. (2006). Data were averaged from three FSD evaluations for each sample (n = 3). Table S3. Secondary structure content of recombinant spider silk assemblies at different pH conditions or soluble monomers directly after ultracentrifugation (bold) determined using FSD of FTIR‐spectra (Figures S5 and S6) according to Hu et al. (2006). For comparison, secondary structure contents of eADF4(C16) fibrils formed at pH 8 in presence of 150 mM KPi were included. Data were averaged from three FSD evaluations for each protein (n = 3). Figure S1. Influence of pH on self‐assembly kinetics of eADF4(C16) in presence of 150 mM KPi adjusted to the indicated pH values (2‐fold negatively charged phosphate). Normalized turbidity evolution at 340 nm indicated decreasing self‐assembly rates and increasing lag times with increasing pH values. Data were averaged over three replicates at each condition (n = 3). Obtained dependencies were fitted using sigmoidal curves (Equation 1). Figure S2. Influence of potassium phosphate (KPi) on self‐assembly of eADF4(Ω16) analyzed using TEM. The investigated KPi‐concentrations were (A) 30 mM, (B) 50 mM, (C) 100 mM, (D i + ii) 150 mM, (E) 200 mM, (F) 300 mM, (G) 400 mM and (H) 500 mM. TEM showed formation of eADF4(Ω16) nanofibrils between 30 mM and 150 mM KPi and particle formation beginning at 150 mM KPi. The orange box highlights the pres [file PRO-35-e70554-s001.docx]

Supporting information

Phosphate- and pH-dependent self-assembly of recombinant spider silk proteins

*Vanessa T. Trossmann^¥,a^, Veronika Hovanová^¥,b-d^, Tim Schiller^a^, Martin Humenik^a^, Erik Sedlák^c-e,*^, Thomas R. Scheibel^a,f-i,*^*

^a^ Department of Biomaterials, Faculty of Engineering Science, University of Bayreuth, Prof.-Rüdiger-Bormann-Str. 1, 95447 Bayreuth, Germany

^b^ FZU - Institute of Physics of the Czech Academy of Sciences, Na Slovance 1999/2, 182 00, Prague, Czech Republic

^c^ Department of Biophysics, Faculty of Science, P.J. Šafárik University, Jesenná 5, 041 54 Košice, Slovakia

^d^ Center for Interdisciplinary Biosciences, Technology and Innovation Park, P.J. Šafárik University, Jesenná 5, 041 54 Košice, Slovakia

^e^ Department of Biochemistry, Faculty of Science, P. J. Šafárik University, Moyzesová 11, 04001 Košice, Slovakia

^f^ Bayreuth Center for Colloids and Interfaces, University of Bayreuth, Universitätsstraße 30, 95447 Bayreuth, Germany

^g^ Bayreuth Center for Molecular Biosciences, University of Bayreuth, Universitätsstraße 30, 95447 Bayreuth, Germany

^h^ Bayreuth Center for Material Science, University of Bayreuth, Universitätsstraße 30, 95447 Bayreuth, Germany

^i^ Bavarian Polymer Institute, University of Bayreuth, Universitätsstraße 30, 95447 Bayreuth, Germany

^¥^ These authors contributed equally.

* Corresponding authors:
E.S. erik.sedlak@upjs.sk

T.R.S. thomas.scheibel@uni-bayreuth.de

**Supporting Tables:**

**Table S1.** Corresponding apparent rate constants (k_app_) and lag times of kinetic measurements. **A)** concerning the data shown in **Figure 2A** for 10 μM eADF4(Ω16) spider silk proteins in presence of different KPi concentrations; **B)** concerning the data shown in **Figure 5A** for 10 μM eADF4(C16) and eADF4(Ω16) spider silk proteins in buffers with different pH values; **C)** concerning the data shown in **Figure S1** for 10 μM eADF4(C16) in KPi at indicated pH.

| **(A)** | | |
| --- | --- | --- |
| **Sample** | **k_app_ (×10⁻³ min⁻¹)** | **lag time (min)** |
| eADF4(Ω16) + 30 mM KPi | 19.6 | 48.2 |
| eADF4(Ω16) + 50 mM KPi | 21.4 | 48.0 |
| eADF4(Ω16) + 75 mM KPi | 28.6 | 32.3 |
| eADF4(Ω16) + 100 mM KPi | 36.1 | 22.7 |
| eADF4(Ω16) + 150 mM KPi | 100.8 | 18.8 |

| **(B)** | | |
| --- | --- | --- |
| **Sample** | **k_app_ (×10⁻³ min⁻¹)** | **lag time (min)** |
| eADF4(C16) pH 3 | - | - |
| eADF4(C16) pH 5 | 14.8 | 69.9 |
|  | | |
| eADF4(Ω16) pH 3 | 18.7 | 17.0 |
| eADF4(Ω16) pH 5 | 18.5 | 17.7 |
| eADF4(Ω16) pH 8 | 16.8 | 47.3 |
| eADF4(Ω16) pH 10 | 17.2 | 228.7 |

| **(C)** | | |
| --- | --- | --- |
| **Sample in 150 mM KPi** | **k_app_ (×10⁻³ min⁻¹)** | **lag time (min)** |
| eADF4(C16) pH 8 | 4.1 | 640.2 |
| eADF4(C16) pH 9 | 2.9 | 1041.3 |
| eADF4(C16) pH 10 | 1.7 | 3199.5 |

**Table S2.** Secondary structure content of recombinant eADF4(Ω16) assemblies at different KPi concentrations determined using FSD of FTIR-spectra (**Figure 1B**) according to Hu et al. (Hu et al., 2006). Data were averaged from three FSD evaluations for each sample (n=3).

| **Protein + Condition** | **β-sheets [%]** | **Random coil [%]** | **α-helices [%]** | **β-turns [%]** | **Side chains [%]** |
| --- | --- | --- | --- | --- | --- |
| eADF4(Ω16) + 30 mM KPi | 40.8 ± 0.6 | 23.0 ± 0.9 | 10.0 ± 0.4 | 19.7 ± 0.7 | 6.6 ± 0.6 |
| eADF4(Ω16) + 50 mM KPi | 44.4 ± 0.5 | 22.9 ± 0.4 | 10.0 ± 0.2 | 18.6 ± 0.4 | 4.1 ± 0.2 |
| eADF4(Ω16) + 100 mM KPi | 43.6 ± 2.2 | 23.1 ± 0.5 | 10.4 ± 0.4 | 19.1 ± 0.3 | 3.8 ± 2.1 |
| eADF4(Ω16) + 150 mM KPi | 42.8 ± 0.4 | 23.0 ± 0.8 | 9.9 ± 0.6 | 19.9 ± 0.3 | 4.4 ± 1.3 |
| eADF4(Ω16) + 200 mM KPi | 43.3 ± 1.0 | 23.4 ± 0.7 | 10.2 ± 0.4 | 19.6 ± 0.1 | 3.5 ± 0.3 |
| eADF4(Ω16) + 500 mM KPi | 40.6 ± 1.0 | 24.4 ± 0.3 | 10.3 ± 0.1 | 20.5 ± 0.4 | 4.3 ± 0.7 |

**Table S3.** Secondary structure content of recombinant spider silk assemblies at different pH conditions or soluble monomers directly after ultracentrifugation (**bold**) determined using FSD of FTIR-spectra (**Figure S5, Figure S6**) according to Hu et al. (Hu et al., 2006). For comparison, secondary structure contents of eADF4(C16) fibrils formed at pH 8 in presence of 150 mM KPi were included. Data were averaged from three FSD evaluations for each protein (n=3).

| **Protein + Condition** | **β-sheets [%]** | **Random coil [%]** | **α-helices [%]** | **β-turns [%]** | **Side chains [%]** |
| --- | --- | --- | --- | --- | --- |
| eADF4(C16), pH 3 | 50 ± 2.7 | 21 ± 1.2 | 7 ± 1.8 | 20 ± 0.5 | 1 ± 0.6 |
| eADF4(C16), pH 5 | 42 ± 3.1 | 17 ± 1.7 | 9 ± 0.2 | 22 ± 1.1 | 10 ± 1.0 |
| **eADF4(C16), pH 8**  **soluble** | **14.0 ± 1.8** | **36.8 ± 4.1** | **16.7 ± 0.7** | **24.6 ± 0.9** | **7.0 ± 3.4** |
| eADF4(C16), pH 8,  150 mM KPi | 51 ± 0.2 | 19 ± 1.4 | 7 ± 3.3 | 22 ± 1.8 | 1 ± 2.4 |
|  | | | | | |
| eADF4(Ω16), pH 3 | 41.0 ± 4.3 | 24.0 ± 1.7 | 10.6 ± 0.4 | 20.6 ± 0.2 | 3.7 ± 2.3 |
| eADF4(Ω16), pH 5 | 42.5 ± 1.4 | 24.2 ± 1.0 | 10.7 ± 0.5 | 19.2 ± 0.6 | 3.5 ± 1.9 |
| **eADF4(Ω16), pH 8**  **soluble** | **23.3 ± 2.8** | **31.2 ± 3.3** | **16.9 ± 2.8** | **23.5 ± 1.8** | **5.1 ± 1.4** |
| eADF4(Ω16), pH 10 | 39.1 ± 3.5 | 23.0 ± 0.2 | 10.5 ± 0.1 | 22.4 ± 1.6 | 5.9 ± 4.3 |

**Supporting Figures:**


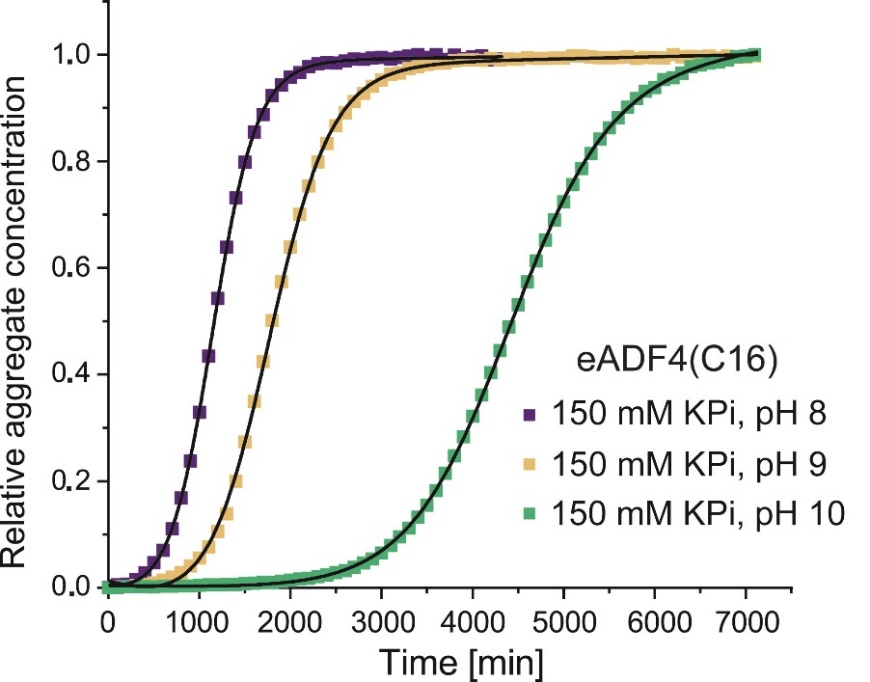


**Figure S1.** Influence of pH on self-assembly kinetics of eADF4(C16) in presence of 150 mM KPi adjusted to the indicated pH values (2-fold negatively charged phosphate). Normalized turbidity evolution at 340 nm indicated decreasing self-assembly rates and increasing lag times with increasing pH values. Data were averaged over three replicates at each condition (n=3). Obtained dependencies were fitted using sigmoidal curves (Eq. 1).


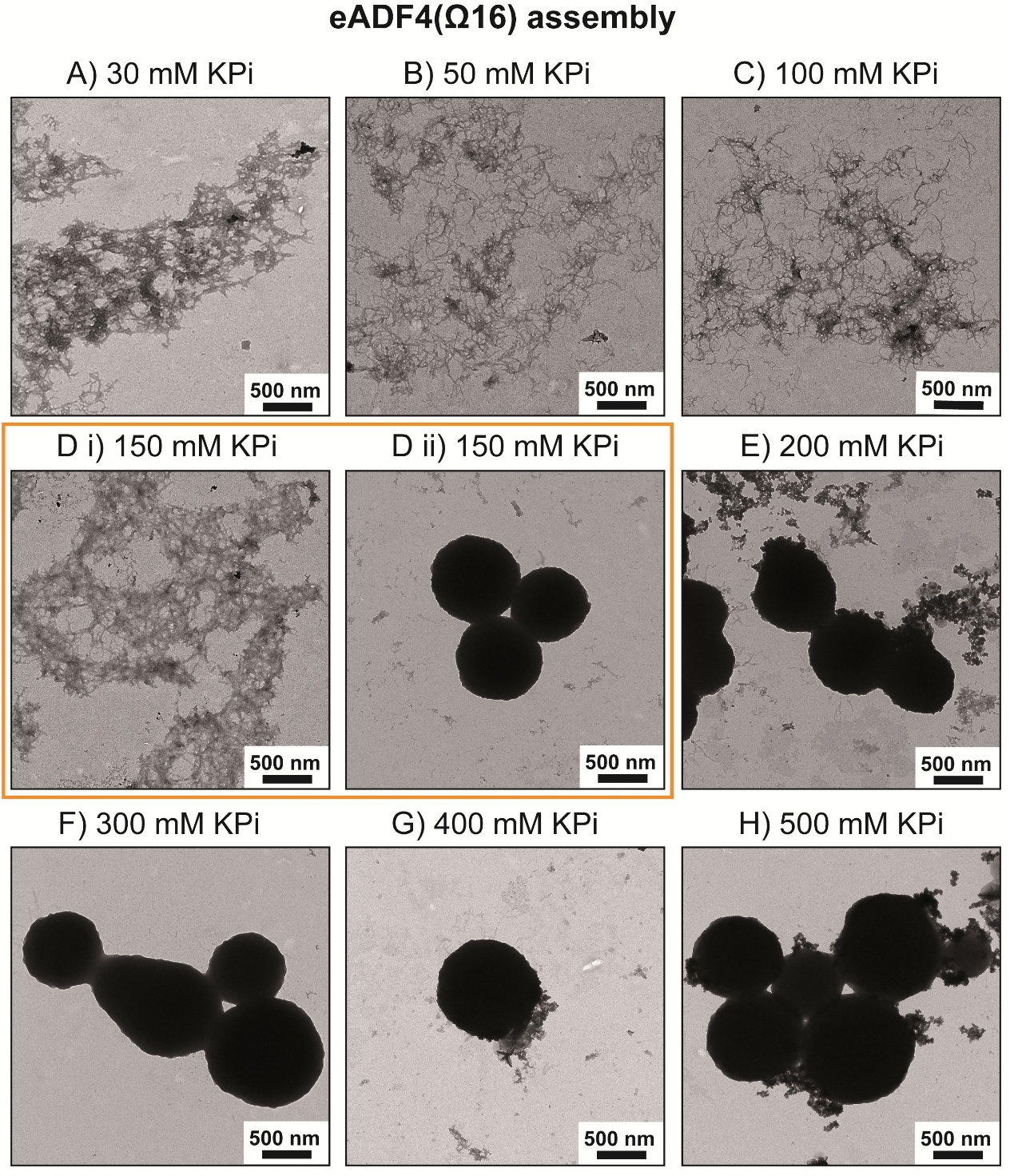


**Figure S2.** Influence of potassium phosphate (KPi) on self-assembly of eADF4(Ω16) analyzed using TEM. The investigated KPi-concentrations were **A**) 30 mM, **B**) 50 mM, **C**) 100 mM, **D i+ii**) 150 mM, **E**) 200 mM, **F**) 300 mM, **G**) 400 mM and **H**) 500 mM. TEM showed formation of eADF4(Ω16) nanofibrils between 30 mM and 150 mM KPi and particle formation beginning at 150 mM KPi. The orange box highlights the presence of both species, nanofibrils and particles, at a KPi-concentration of 150 mM.


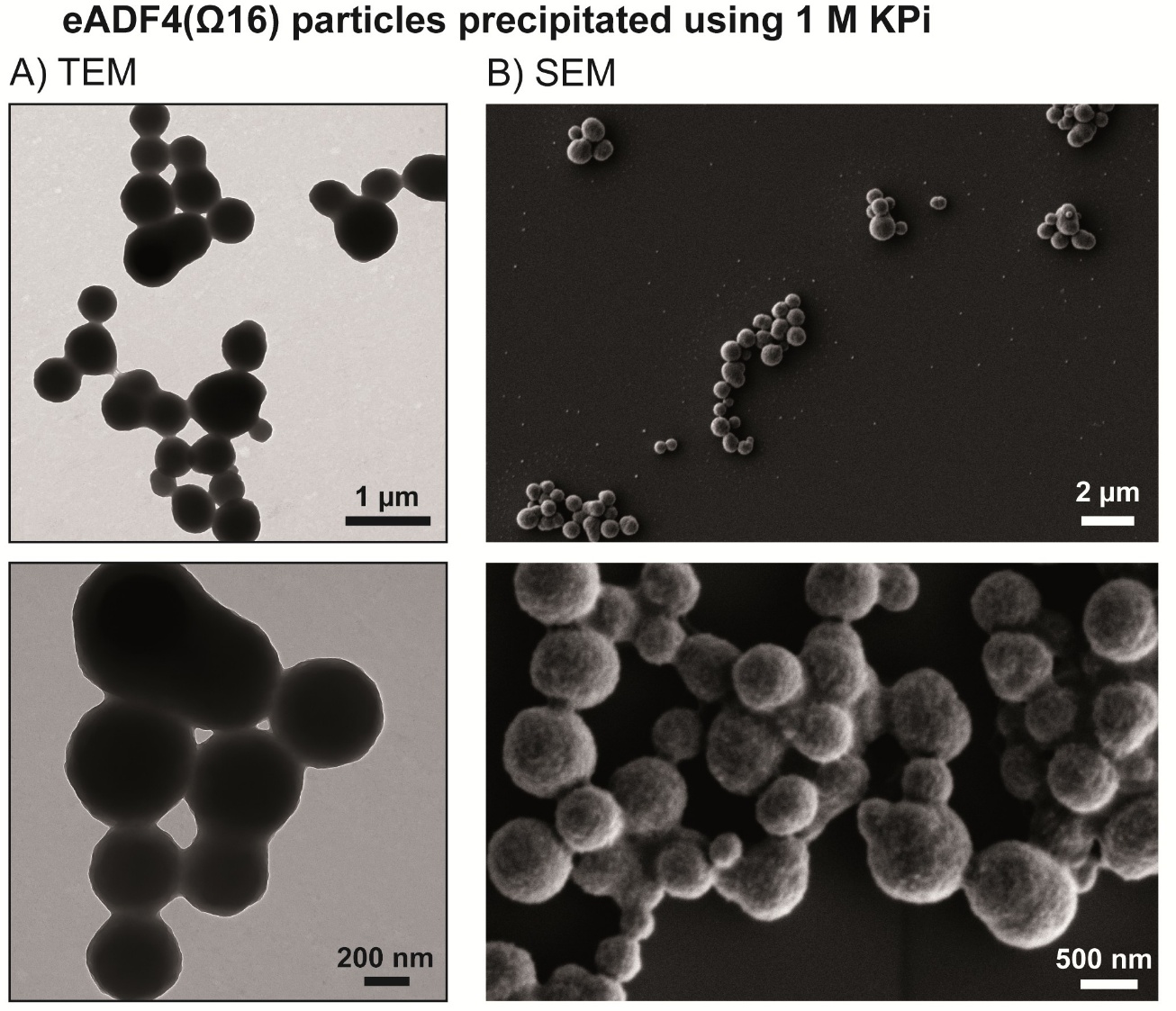


**Figure S3.** TEM **(A)** and SEM **(B)** of air-dried, spherical eADF4(Ω16) particles precipitated at 1 M KPi using a salting-out procedure.


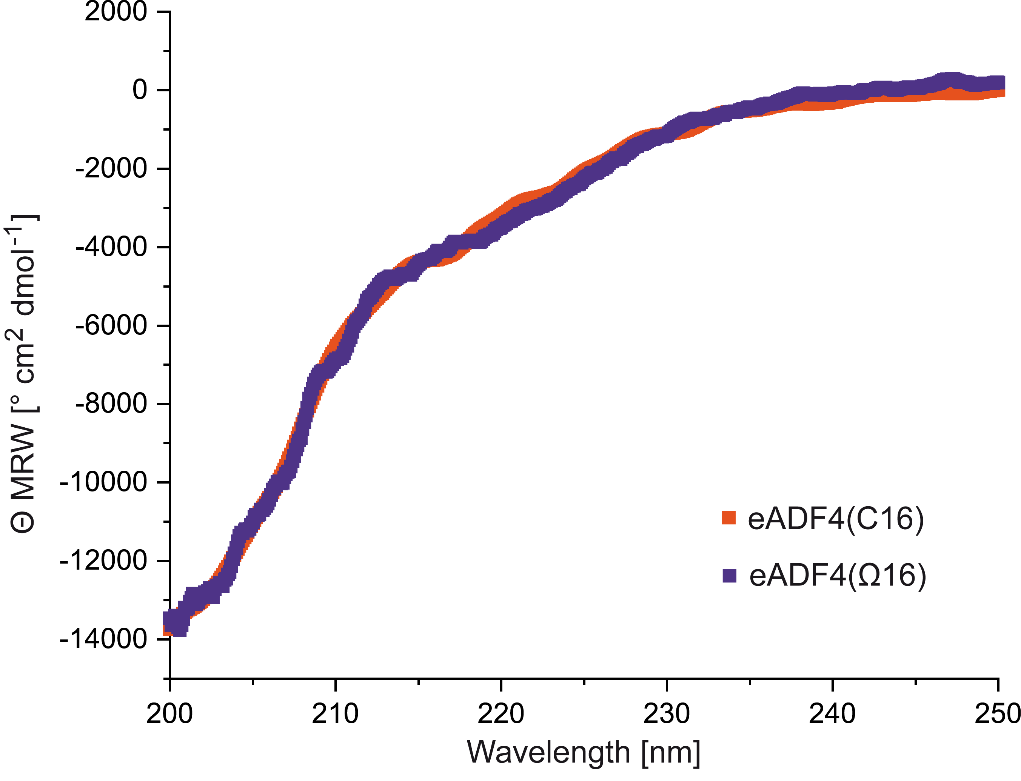


**Figure S4.** Far-UV circular dichroism spectra indicate a random coil secondary structure of soluble eADF4(C16) and eADF4(Ω16) after ultracentrifugation. Data were averaged over five replicates at each condition (n=5).


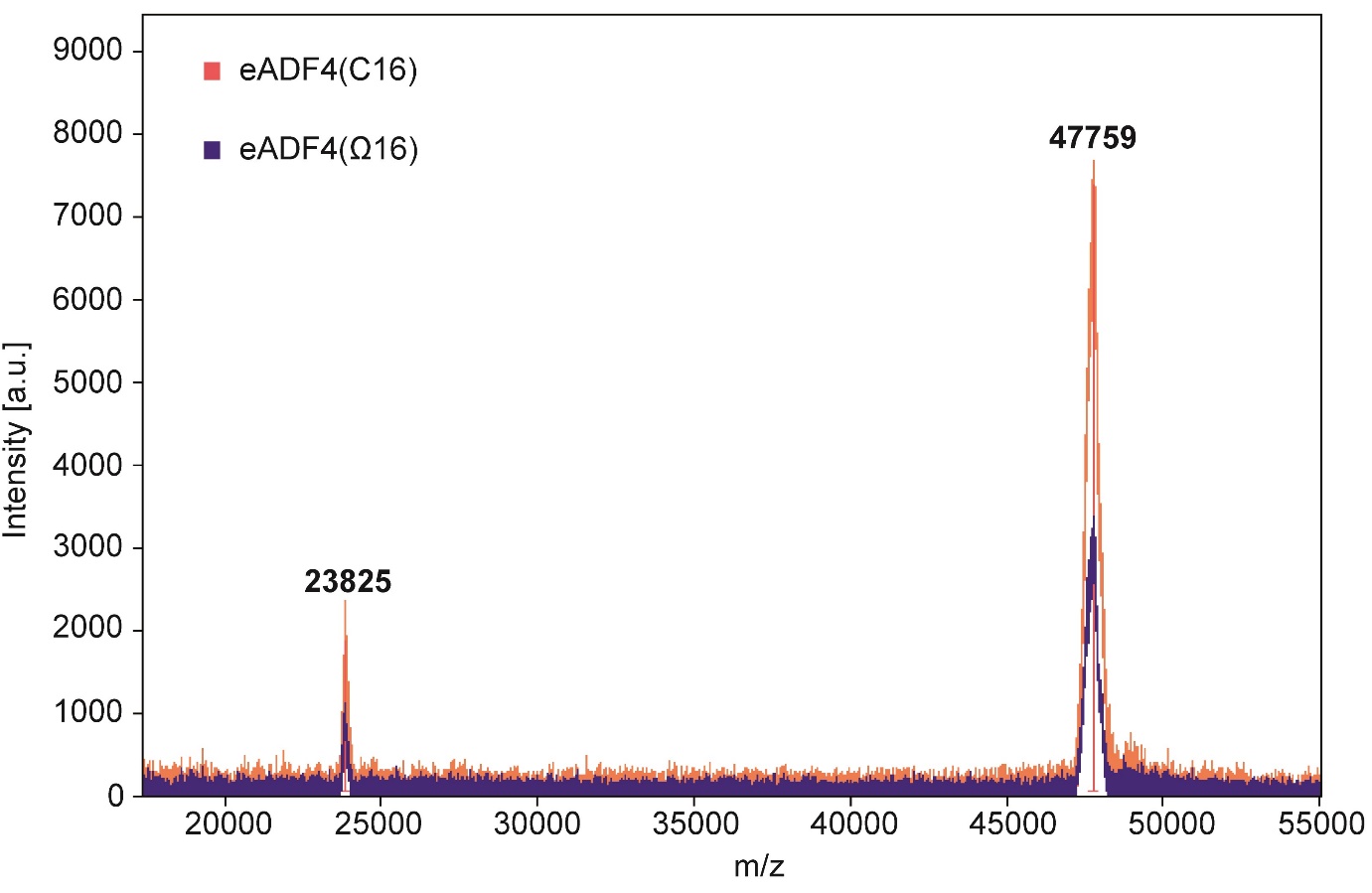


**Figure S5.** Maldi-ToF spectra confirm the theoretical MW of recombinant eADF4(C16) (theoretical MW: 47,698 Da) and eADF4(Ω16) (theoretical MW: 47,683 Da).


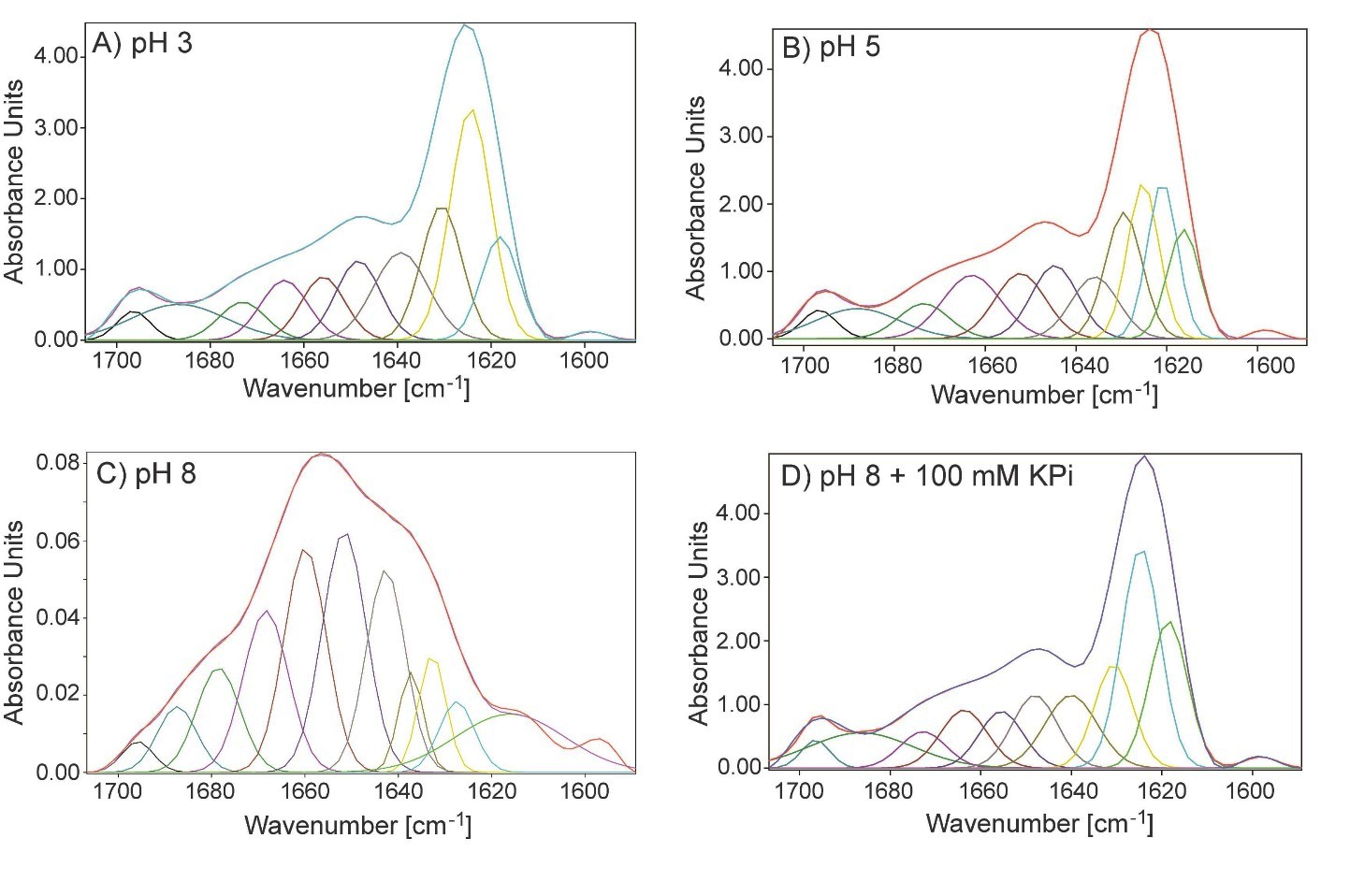


**Figure S6.** Exemplary FSD-analysis of the Amide I band of eADF4(C16)-based assemblies formed at pH 3 (A) and 5 (B), soluble protein at pH 8 (C) and fibrils formed in presence of 150 mM KPi (D) to determine secondary structure content (n=3).


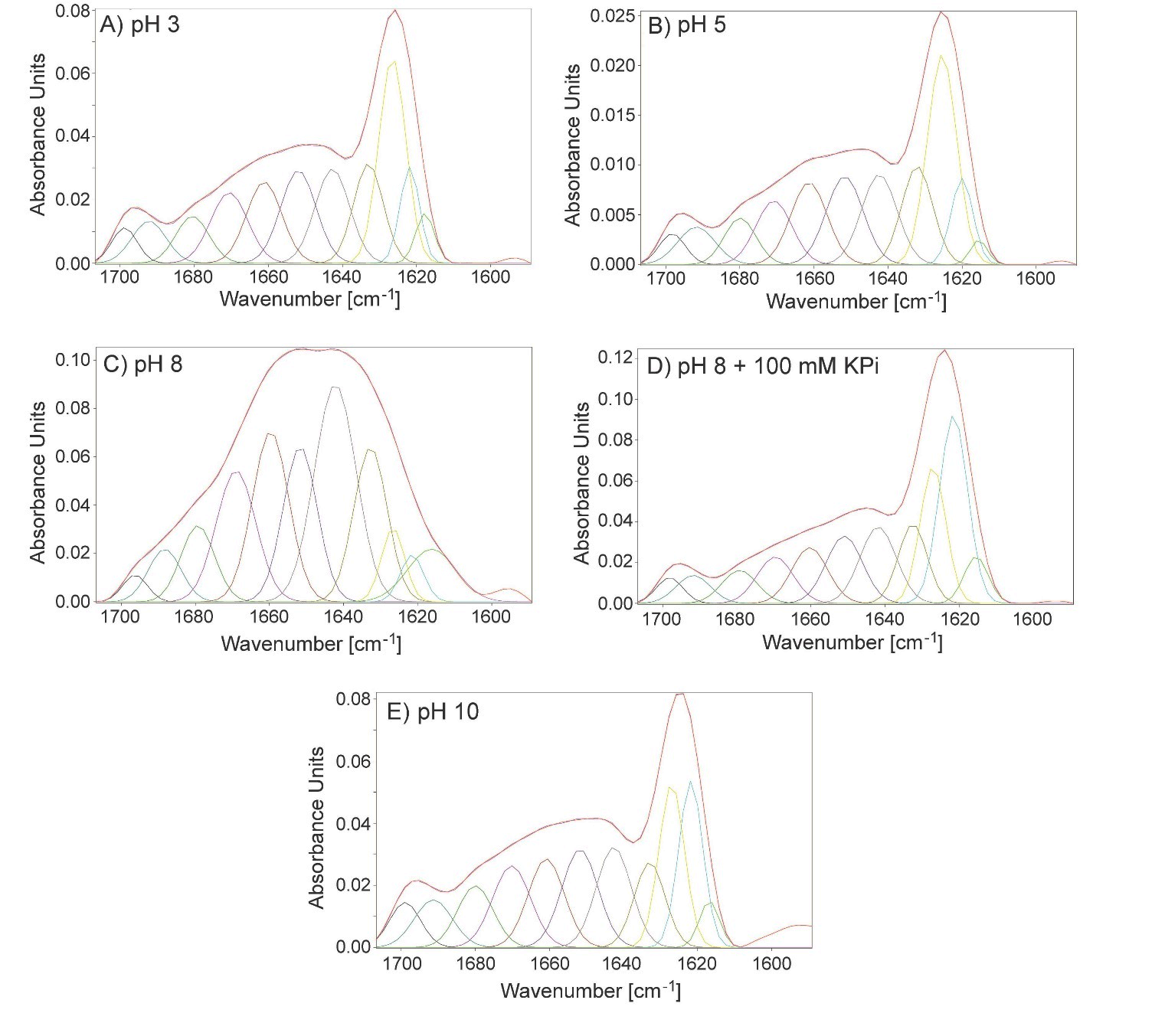


**Figure S7.** Exemplary FSD-analysis of the Amide I band of eADF4(Ω16)-based assemblies formed at pH 3 (A), 5 (B) and 10 (E), soluble protein at pH 8 (C) and fibrils formed in presence of 100 mM KPi (D) to determine secondary structure content (n=3).
